# HLA allele profiling and association in Thai patients with confirmed beta-lactam hypersensitivity: an NGS-based analysis

**DOI:** 10.3389/fphar.2026.1782094

**Published:** 2026-07-10

**Authors:** Lalita Lumkul, Prapasri Kulalert, Mongkhon Sompornrattanaphan, Thanachit Krikeerati, Supharat Suvichapanich, Patcharawadee Thongkumkoon, Pitiporn Noisagul, Chamard Wongsa, Torpong Thongngarm, Wannada Laisuan, Dumnoensun Pruksakorn, Padcha Pongcharoen, Sira Nanthapisal, Ratchaya Lertnawapan, Phichayut Phinyo

**Affiliations:** 1 Center for Clinical Epidemiology and Clinical Statistics, Faculty of Medicine, Chiang Mai University, Chiang Mai, Thailand; 2 Center of Multidisciplinary Technology for Advanced Medicine (CMUTEAM), Faculty of Medicine, Chiang Mai University, Chiang Mai, Thailand; 3 Department of Clinical Epidemiology, Faculty of Medicine, Thammasat University, Pathumthani, Thailand; 4 Department of Pediatrics, Division of Allergy and Clinical Immunology, Faculty of Medicine, Thammasat University, Pathumthani, Thailand; 5 Division of Allergy and Clinical Immunology, Department of Medicine, Faculty of Medicine Siriraj Hospital, Mahidol University, Bangkok, Thailand; 6 Center of Research Excellence in Allergy and Immunology, Faculty of Medicine Siriraj Hospital, Mahidol University, Bangkok, Thailand; 7 Department of Biochemistry, Faculty of Pharmacy, Mahidol University, Bangkok, Thailand; 8 Department of Medicine, Division of Allergy Immunology and Rheumatology, Faculty of Medicine, Ramathibodi Hospital, Mahidol University, Bangkok, Thailand; 9 Department of Orthopaedics, Faculty of Medicine, Chiang Mai University, Chiang Mai, Thailand; 10 Department of Internal Medicine, Division of Dermatology, Faculty of Medicine, Thammasat University, Rangsit Campus, Pathumthani, Thailand; 11 Department of Internal Medicine, Division of Allergy Immunology and Rheumatology, Faculty of Medicine, Thammasat University, Pathumthani, Thailand; 12 Department of Biomedical Informatics and Clinical Epidemiology (BioCE), Faculty of Medicine, Chiang Mai University, Chiang Mai, Thailand

**Keywords:** allergy, antibiotics, beta-lactam hypersensitivity reactions, genetic association, human leukocyte antigen, HLA

## Abstract

**Background:**

Beta-lactams (BLs) are the most common reported antibiotics associated with hypersensitivity reactions (HSRs). However, evidence for human leukocyte antigen (HLA) associations with beta-lactam hypersensitivity reaction (BL-HSR) remains limited and population-specific, particularly in the Thai population.

**Objectives:**

This study aims to explore HLA profiles and investigate associations between HLA class I and II alleles and confirmed BL-HSR in Thai adults, across immediate reactions (IR), non-immediate reactions with mild skin eruption (NIR-mild), and severe cutaneous adverse reactions (SCARs), and to contextualize these findings with regional data.

**Methods:**

This study was based on a case-conrtol design with exploratory analysis approach. For cases, we enrolled adult patients with confirmed BL-HSR from three Thai tertiary hospitals. IR and NIR-mild cases were confirmed by positive skin testing and/or drug provocation testing, while SCAR cases were clinically confirmed. Their blood samples underwent whole exome sequencing, and HLA typing was performed. For control group, we derived allele frequency data of HLA genes in Thai populations from previous nationwide studies previous nationwide studies and databases. HLA allele associations were assessed by Fisher’s exact test with Bonferroni correction. Subgroup analyses were conducted based on reaction types and culprit drugs. Meta-analyses of specific HLA alleles from Thai and Asian BL-HSR studies were carried out using random-effects model.

**Results:**

Thirty-seven patients were included (62.16% IR, 21.62% NIR-mild, 16.22% SCARs). Association analyses identified candidate susceptibility signals for BL-HSR including HLA-DQA1*01:04 (odds ratio (OR) 8.79; 95% confidence interval (CI) 2.01–29.87; adjusted p-value 0.029) and HLA-DQA1*05:05 (OR 5.03; 95% CI 1.66–12.74; adjusted p-value 0.029). HLA-DQA1*05:05 showed stronger association in IR (OR 6.96; 95% CI 2.01–19.25; adjusted p-value: 0.018). Penicillin-induced HSR were potentially associated with HLA-C*07:02, and β-lactamase inhibitor with HLA-DQA1*01:04. However, these signals were not robust in subsequent sensitivity analyses. Meta-analyses provided contextual support for previously reported high-risk alleles in BL-induced SCARs.

**Conclusion:**

HLA-DQA1*01:04 and HLA-DQA1*05:05 represent candidate susceptibility signals for BL-HSR in Thai adults, with phenotype- and drug-specific patterns, which require independent validation. These findings, given relatively small sample size and use of population-based controls, may suggest basis for future immunogenetic risk stratification with validation in larger, phenotype-specific populations.

## 1 Introduction

Beta-lactam antibiotics (BLs) including penicillins, cephalosporins, carbapenems, monobactams, and beta-lactamase inhibitors, are among the most prescribed antimicrobial agents worldwide, accounting for nearly 65% of antibiotic use (Thakuria and Lahon, 2013). Hypersensitivity reactions (HSR) to BLs are frequently reported, affecting approximately 8%–15% of treated individuals (Sousa-Pinto et al., 2017; Zhou et al., 2016). These reactions range from mild skin eruptions (e.g., urticaria, maculopapular exanthema (MPE)) to life-threatening anaphylaxis and severe cutaneous adverse reactions (SCARs), including Stevens-Johnson syndrome (SJS), toxic epidermal necrolysis (TEN), and drug reaction with eosinophilia and systemic symptoms (DRESS). Specifically, among antibiotic-induced SCARs, 12%–44% are attributed to BLs, making them a major cause of drug-related morbidity (Hsu et al., 2016; Micheletti et al., 2018).

The immunopathogenesis of beta-lactam hypersensitivity reaction (BL-HSR) depends on the timing and mechanism of immune activation (Shenoy et al., 2019). Immediate reactions (IR) occur within minutes to 1–6 h and are typically mediated by drug-specific IgE antibodies that trigger mast cell degranulation, leading to urticaria, angioedema, or anaphylaxis. In contrast, non-immediate reactions (NIR) occur more than 1 h to days after exposure and are mediated by drug-specific T lymphocytes recognizing drug–peptide complexes presented by human leukocyte antigen (HLA) molecules, resulting in maculopapular exanthems or SCARs (Ariza et al., 2015). Several hypotheses have been proposed to explain these immune hypersensitivity pathways include the hapten/pro-hapten, pharmacological interaction (p-i), and danger hypotheses (Gerberick et al., 2009; Pichler, 2002; Matzinger, 1994).

From a preventive perspective, HLA-based genetic testing represents an emerging strategy for predicting drug hypersensitivity (Kloypan et al., 2021). Landmark pharmacogenomic discoveries, such as HLA-B*15:02 for carbamazepine-induced SJS/TEN and HLA-B*57:01 for abacavir-induced SCARs, have led to routine pre-prescription genetic testing in several Asian countries, supported by The Clinical Pharmacogenetics Implementation Consortium (CPIC) and FDA guidelines (Zhou et al., 2021; Martin et al., 2014; Phillips et al., 2018; TUSFaD, 2016). However, the clinical utility of HLA testing for BLs remains limited due to weaker associations, low positive predictive values, high number needed to test (Jeimy et al., 2020). Furthermore, differences in allele frequencies among populations likely play a key role in this variability. While HLA-B*55:01 has been linked to penicillin allergy in Europeans, Asian studies have reported associations with HLA-C*04:06, HLA-C*08:01, and HLA-B*48:01 in pediatric and adult BL-HSR cohorts (Krebs et al., 2020; Nicoletti et al., 2021; Singvijarn et al., 2021; Wang CC. et al., 2024; Lumk et al., 2023). This ethnic variation in HLA alleles highlights the need for population-specific pharmacogenomic studies.

In Thailand, BLs remain the leading cause of antibiotic hypersensitivity, with penicillin and cephalosporin accounting for 24.6% and 16.4% of antibiotic HSRs, respectively. Likewise, among children with adverse drug reactions, BL were the most frequently implicated antibiotics, particularly amoxicillin, ceftriaxone and cephalexin (Chongpison et al., 2023; Sangasapasviliya et al., 2010; Tantikul et al., 2008), yet genetic evidence remains limited. Only one study identified HLA-C*04:06 and HLA-C*08:01 as potential risk alleles in children (Singvijarn et al., 2021), whereas adult studies on BL-induced SCARs have yielded non-significant associations (Wattanachai et al., 2023). To address this gap, the present study aimed to investigate HLA allele associations with BL-HSR in Thai adults, including IR, NIR with mild skin eruption (NIR-mild), and SCAR phenotypes, to demonstrate potential immunogenetic risk factors underlying BL allergy in the Thai population and to provide foundational evidence for the future implementation of pharmacogenomic screening.

## 2 Materials and methods

### 2.1 Study population

This genetic association study employed a case-control design. The study protocol was approved by the institutional review board**s** from the Faculty of Medicine, Chiang Mai University; Faculty of Medicine Siriraj Hospital, Mahidol University; the Faculty of Medicine Ramathibodi Hospital, Mahidol University; and the Faculty of Medicine, Thammasat University. Adult patients (≥18 years) with confirmed beta-lactam hypersensitivity reactions (BL-HSRs) were recruited from three tertiary care hospitals: (1) Siriraj Hospital, (2) Ramathibodi Hospital, and (3) Thammasat University Hospital. Written informed consent was obtained from all patients before enrollment.

Patients were defined as cases if they met one of the following criteria: (i) patients with a clinical diagnosis of severe cutaneous adverse reaction (SCARs), including Stevens–Johnson syndrome (SJS), toxic epidermal necrolysis (TEN), SJS/TEN, drug reaction with eosinophilia and systemic symptoms (DRESS), or acute generalized exanthematous pustulosis (AGEP), with or without lymphocyte transformation test, with diagnosis made by an allergist and/or dermatologist based on the clinical criteria [e.g., for SJS/TEN (Brockow et al., 2019), for DRESS (Calle et al., 2023), and for AGEP (Parisi et al., 2023)]; or (ii) patients with clinically suspected drug hypersensitivity who completed testing and had at least one positive result for skin prick test, intradermal test, or drug provocation test according to institutional protocols. Reaction onset was categorized as either immediate (within ≤1 h of the last dose) or non-immediate (more than 1 h after the last dose). Patients were excluded if they had unretrievable medical history (i.e., patients who did not have allergic test history, unknown drug groups), denied to participation or had type II and III hypersensitivity, e.g., acute interstitial nephritis, drug-induced liver injury, organ-specific reactions, or serum sickness. Patients’ information was collected including basic demographic (e.g., age, sex, weight, height, underlying allergic diseases), history of previous hypersensitivity reactions (e.g., time of last reaction occurred, number of episodes, reaction symptoms, treatment required of the reaction), allergic test results (e.g., type of the test, culprit drugs). Another drug allergy or co-allergies was collected from previous clinical history or by patient-report questionnaires. Multiple drug hypersensitivity (MDH) test was not systematically applied in this cohort.

The control group was defined as the Thai general population. HLA data for this group were retrieved from previous publications and from the Allele Frequency Net Database, as detailed in the subsequent sections.

### 2.2 Sample preparation

Peripheral blood samples were collected from patients and were transferred to the Faculty of Medicine, Chiang Mai University, under 4 °C storage temperature. Then, peripheral blood mononuclear cells (PBMCs) were collected, and stored at −80 °C at the faculty’s repository unit. Genomic DNA was extracted using the QIAamp DNA Blood Kit for Genomic DNA Extraction (QIAGEN, Germany) according to the manufacturer’s protocol. The purity and concentration of the extracted DNA were determined by NanoDrop® (Thermo Fisher Scientific, United States). The extracted DNA was stored at 4 °C before further analysis.

### 2.3 Whole exome sequencing

The obtained genomic DNA samples were sent to Macrogen (Seoul, South Korea) for Illumina whole exome sequencing (WES). The standard protocols for SureSelect Human all exon V8 with Illumina paired-end adapters were used for library preparation from the fragmented genomic DNA. Sequencing libraries were constructed and WES was performed using Illumina NovaSeq technology with 150-bp paired-end reads. A mean target coverage of 200X was achieved for raw data per sample.

### 2.4 HLA typing

Prior to downstream analyses, the raw sequence reads of each sample were checked for quality using FastQC version 0.12.1 to evaluate base quality, adapter contamination, and GC distribution. Raw reads were then processed with fastp version 1.0.0 to perform adapter trimming and poly-G tail trimming as appropriate. Post-trimming quality was evaluated with FastQC, and adapter content was re-checked on the cleaned FASTQs. If residual adapter signal was detected, reads were re-processed accordingly before downstream analyses.

For HLA typing, HLA class I and class II alleles were typed using HLA-HD version 1.7.1 (Kawaguchi et al., 2017; Kawaguchi et al., 2018; Kawaguchi and Matsuda, 2020). Briefly, raw paired-end FASTQ files were processed following the HLA-HD pipeline. Each sample’s reads were first decompressed and aligned to the IMGT/HLA reference genome using Bowtie2 (v2.5.4). Mapped reads were extracted with SAMtools (v1.15.1) and converted back to paired FASTQ format using Picard SamToFastq. HLA typing was performed with HLA-HD using default parameters, trimming rate 0.95, and minimum read length 100. The reference dictionary and allele frequency data provided by the developer were used for allele assignment. Output files containing allele calls for HLA class I (A, B, C) and class II (DQA1, DQB1, DRB1) genes were collected. The HLA alleles at two-field (four-digit) or three-field (six-digit) resolutions were obtained. The three-field alleles were combined to two-field resolutions for comparative purposes. Then, allele frequencies for each individual were counted manually for descriptive analysis and the association test.

### 2.5 Haplotype estimation

Haplotype estimation was performed using the haplo. stats package in R (version 1.9.7) (Schaid et al., 2002). Expectation–Maximization (EM) algorithm implemented in the haplo. em function was used to estimate haplotype frequencies across multiple locus combinations with unphased data. Analyses were conducted for different sets of loci. The resulting haplotypes were ranked by their estimated frequency, and results for each locus combination, from two loci to seven loci, were exported for association testing.

### 2.6 Thai population reference datasets

Allele frequencies (AF) of Thai population were obtained from published genotyping dataset**s** of a nationwide Thai cohort with no history of cutaneous adverse drug reaction (n = 470) (Satapornpong et al., 2020), and from a dataset of unrelated healthy donors enrolled in a dengue vaccine clinical trial in Thailand (n = 334) (Geretz et al., 2018). Additional allele frequency data were retrieved from the Allele Frequency Net Database (AFND), which included all available HLA genotyping results from Thai cohorts: Thailand cohort (n = 142) (Thailand, 2002), Thai Northeast (n = 66) (Chandanayingyong et al., 1994), Thai Northeast pop 2 (n = 400) (Romphruk et al., 2010), Thailand Kamphaeng Phet (n = 97) (Maneemaroj et al., 1997), Thailand North Dai Lue (n = 96) (Chandanayingyong et al., 1994; Chandanayingyong et al., 1997), Thailand Bangkok pop 2 (n = 50) (Nathalang et al., 1999), Thailand Bangkok pop 3 (n = 187) (Sirikong et al., 2002), Thailand pop 3 (n = 49) (Pimtanothai et al., 2002), Thailand pop 2 (n = 124) (Wongsurawat et al., 2006), Thailand Bangkok (n = 104) (Chandanayingyong et al., 1994), and Thailand East Chanthaburi Khmer (n = 83) (Chandanayingyong et al., 1994). Data from a total of 1,842 individuals were included and frequencies were harmonized to two-field resolution to match across studies. For each HLA locus, allele frequency was converted to allele count using the formula: (allele count = frequency × total sample size per cohort), and the resulting values were rounded to the nearest integer. Allele counts from all cohorts were aggregated to form a pooled control dataset. The total number of control samples varied across HLA loci, as some cohorts did not report all genes.

### 2.7 Population structure distribution

To assess the population structure of HLA alleles in our study cohorts, principal component analysis (PCA) was performed using AF data from each population cohort. All AF were harmonized by matching HLA loci between the case and control populations. Control populations with two or more missing HLA loci were excluded. In the remaining populations, HLA loci with missing AF data in two or more populations were also excluded. The retained HLA loci were then matched with the reference datasets, which included representative populations from diverse genetic backgrounds. These reference datasets comprised gold-standard populations from AFND (Gonzalez-Galarza et al., 2020): North America (Mexico, n = 218) (Barquera et al., 2020), Europe (from Germany (Seitz et al., 2021) and Kosovo, population ID 3740), South Asia (Sri Lanka, n = 714) (Grifoni et al., 2018), and East Asia (Hong Kong, n = 5,266) (Kwok et al., 2020). The AF data were scaled prior to PCA and visualized.

### 2.8 Sample size calculation

Sample size estimation was based on a comparison of allele frequencies between patients with beta-lactam hypersensitivity and control data from a published population-wide study of Thai HLA alleles which comprised 470 unrelated healthy individuals (Satapornpong et al., 2020). Because adult Thai BL-HSR genetic data were limited, this calculation was based on the best available Thai BL HLA association data at the time. The number of cases required was calculated using a two-sample comparison of proportions. The control allele frequencies were compared with published frequencies from Thai children with BL-HSR (Singvijarn et al., 2021). Two significant HLA alleles were selected as parameters for the sample size estimation: HLA-B*48:01 and HLA-C*04:06. For HLA-B*48:01, the reported allele frequencies were 0.0043 in controls and 0.07 in allergic cases, indicating that approximately 37 patients would be required to achieve 80% statistical power at a two-sided α of 0.05. For HLA-C*04:06, with allele frequencies of 0.01 in controls and 0.10 in cases, an estimated 31 patients would be needed to achieve the same level of power and significance. Therefore, the minimum sample size required was set at 37 patients.

### 2.9 Statistical analysis

All statistical analyses were performed in Stata 16.0 (StataCorp, College Station, TX, United States). Clinical characteristics were summarized using descriptive statistics. Mean and standard deviation (SD) were used to summarize continuous variables for data with normal distribution; otherwise, median and interquartile range (IQR) were used as appropriate. Frequency and percentage were used to describe categorical data.

Association analysis at the allele level and haplotype level was assessed using Fisher’s exact test. P-values were adjusted for multiple comparisons using Bonferroni correction per each HLA locus. Odds ratios (ORs) were calculated with their corresponding 95% confidence intervals (95% CI). Subgroup analyses were performed by reaction types and culprit drugs and stratification analysis in patients with co-allergies were conducted. Sensitivity analysis was conducted in alleles with more than 5% allele frequency (either in case or control), and in control subjects indicating no history of cutaneous adverse drug reactions (CADRs) (Satapornpong et al., 2020).

Meta-analysis was performed using our data and available published sources, in particular patient subgroups. Pooled effect size was estimated from reported allele carriers using a random-effects model with maximum likelihood estimation. Heterogeneity was assessed using I-square.

## 3 Results

### 3.1 Clinical characteristics

**FIGURE 1:**
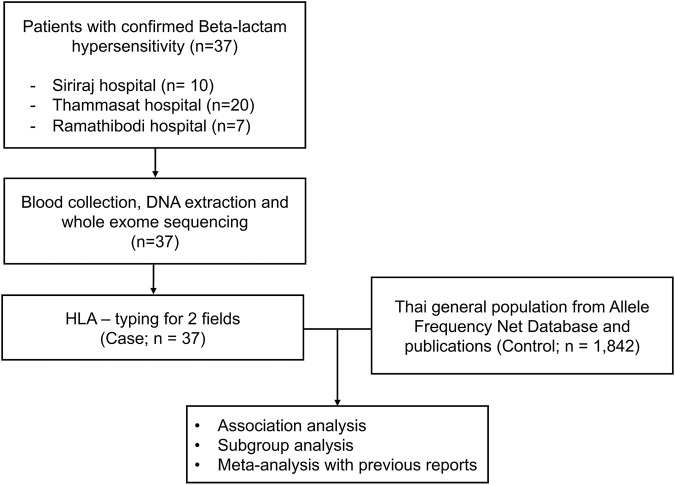
Study flow diagram.

A total of 37 patients with confirmed BL-HSR were recruited from three tertiary care hospitals and underwent sample collection for further analysis (Figure 1). Table 1 summarizes patient demographics and underlying diseases. Of these, 78.4% were female with a mean age of 47.1 (SD 13.8) years. Most of the patients (62.2%) had immediate reaction (IR), of which urticaria (39.1%) and anaphylaxis (39.1%) were primarily observed. NIR-mild occurred in 21.6% of patients, with maculopapular exanthema (50.0%) as the predominant manifestation, while SCARs were observed in 16.2% of patients.

**TABLE 1:** Characteristics of patients (n = 37).

Characteristics (n,%)	All patients (n = 37)	Immediate reaction patients (n = 23)	Non-immediate reaction with mild skin eruptions patients (n = 8)	SCARs patients (n = 6)
Female	29 (78.38)	19 (82.61)	6 (75.00)	4 (66.67)
Age at confirmed hypersensitivity (years), mean (SD)	44.11 (14.15)	42.00 (14.98)	43.88 (11.00)	52.50 (13.43)
BMI (kg/m^2^) mean (SD)	24.25 (3.59)	24.44 (3.91)	24.13 (2.38)	23.69 (4.16)
Age at first episode (years), mean (SD)	37.81 (16.31)	36.32 (16.49)	31.13 (12.22)	52.17 (13.50)
Culprit drugs				
Penicillin	17 (45.95)	10 (43.48)	7 (87.50)	0
Cephalosporin	12 (32.43)_	8 (34.78)	0	4 (66.67)
Beta-lactamase inhibitor	8 (21.62)	5 (21.74)	1 (12.50)	2 (33.33)
Underlying allergic diseases				
Asthma	5 (13.51)	3 (13.04)	1 (12.50)	1 (16.67)
Allergic rhinitis	17 (45.95)	13 (56.52)	3 (37.50)	1 (16.67)
Atopic dermatitis	5 (13.51)	2 (8.70)	1 (12.50)	2 (33.33)
Urticaria	6 (16.22)	4 (17.39)	1 (12.50)	1 (16.67)
Food allergy	12 (32.43)	8 (34.78)	2 (25.00)	2 (33.33)
Another drug allergy	19 (51.35)	13 (56.52)	6 (75.00)	0
Fluoroquinolone	4 (10.81)	2 (8.70)	2 (25.00)	0
Sulfonamides	4 (10.81)	3 (13.04)	1 (12.50)	0
Nitroimidazoles	1 (2.70)	0	1 (12.50)	0
Lincosamides	2 (5.41)	1 (4.35)	1 (12.50)	0
Glycopeptides	1 (2.70)	0	1 (12.50)	0
Chloramphenicol	1 (2.70)	0	1 (12.50)	0
Mupirocin	1 (2.70)	0	1 (12.50)	0
NSAIDs	7 (18.92)	6 (26.09)	1 (12.50)	0
Antiepileptic drugs	1 (2.70)	0	1 (12.50)	0
Antispasmodic drugs	2 (5.41)	2 (8.70)	0	0
Others	5 (13.51)	4 (17.39)	1 (12.50)	0
Reaction symptoms				
Anaphylaxis with or without shock	10 (27.03)	9 (39.13)	1 (12.50)	0
Cutaneous reactions	​	​	​	​
Angioedema	8 (21.62)	5 (21.74)	3 (37.50)	0
Urticaria	9 (24.32)	9 (39.13)	0	0
Urticaria and angioedema	4 (10.81)	3 (13.04)	1 (12.50)	0
Fixed drug eruption	1 (2.70)	0	1 (12.50)	0
Maculopapular exanthema	4 (10.81)	0	4 (50.00)	0
SCARs	​	​	​	​
SJS, SJS overlap	1 (2.70)	-	-	1 (16.67)
AGEP	3 (8.11)	-	-	3 (50.00)
DRESS	2 (5.41)	-	-	2 (33.33)
Number of reaction episodes	​	​	​	​
1 episode	24 (64.86)	15 (65.22)	4 (50.00)	5 (83.33)
>1 episodes	13 (35.14)	8 (34.78)	4 (50.00)	1 (16.67)
Treatment required of the reaction	​	​	​	​
No treatment	9 (24.33)	6 (26.09)	3 (37.50)	0
Outpatient visit	20 (54.05)	15 (65.22)	4 (50.00)	1 (16.67)
In patient admission	8 (21.62)	2 (8.70)	1 (12.50)	5 (83.33)

Abbreviations: AGEP, acute generalized exanthematous pustulosis; BMI, body mass index; DRESS, drug reaction with eosinophilia and systemic symptoms; NSAIDs, nonsteroidal anti-inflammatory drugs; SCARs, severe cutaneous adverse reactions; SJS, Stevens–Johnson syndrome.

Penicillins were the most frequent culprit drug group and were commonly found in both IR and NIR-mild, whereas 66.7% of SCARs were attributed to cephalosporins. Allergic rhinitis was the most frequent underlying allergic condition (46.0%), and approximately 51.4% of patients reported co-allergy to other drugs, which were observed in 56.5% of IR and 75.0% of NIR-mild patients. The most commonly reported additional drug allergies involved antibiotics (i.e., ciprofloxacin, clindamycin, mupirocin, vancomycin, co-trimoxazole), which were identified in 13 patients (35.1%), followed by nonsteroidal anti-inflammatory drugs (NSAIDs; i.e., ibuprofen, naproxen, aspirin), reported in seven patients (18.9%). Other drugs include statin, prednisolone, fentanyl, propofol, pioglitazone (Table 1).

### 3.2 HLA allele frequencies in patients with BL-HSR

**FIGURE 2:**
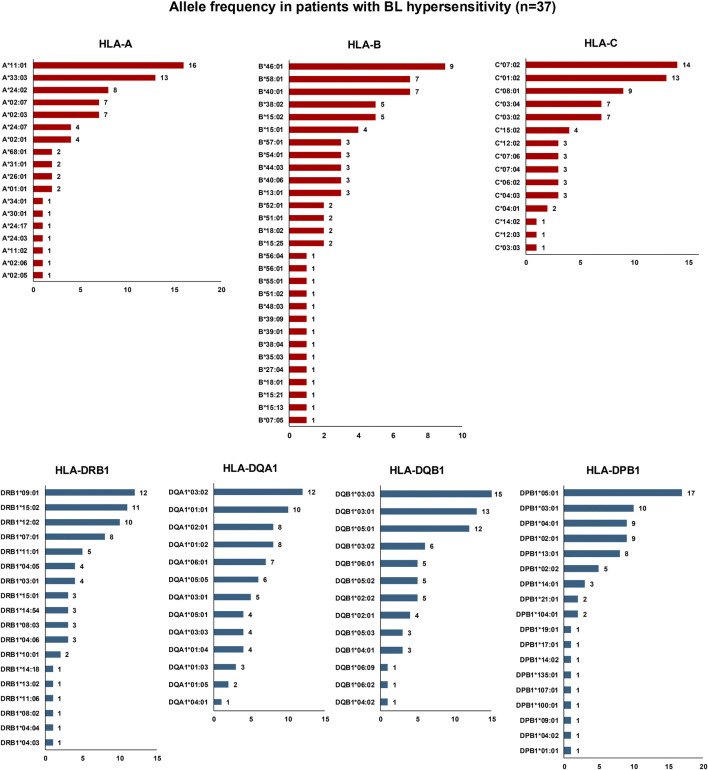
Allele frequency of HLA types found in patients with BL-HSR (n = 37).

Among 37 BL-HSR cases (2n = 74), the number of alleles found in each HLA locus was identified as follows: HLA-A 18 alleles, HLA-B 29 alleles, HLA-C 15 alleles, HLA-DRB1 18 alleles, HLA-DQA1 13 alleles, HLA-DQB1 13 alleles, and HLA-DPB1 18 alleles (Supplementary Tables 1, 2). The identified alleles and their frequencies were illustrated in Figure 2.

**FIGURE 3:**
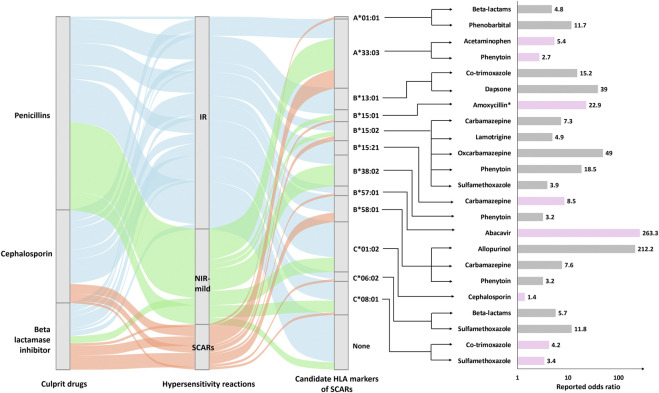
Number of patients with different types of reactions, culprit drugs and their SCARs markers identified. IR, immediate reaction; NIR-mild, non-immediate reaction with mild skin eruption.

Within these cases, several HLA alleles which were previously reported as candidate markers of SCARs in the Thai and Asian populations were identified (Li et al., 2025). The most frequently observed allele was HLA-A*33:03, detected in 6 IR cases, 2 NIR-mild cases, and 3 SCAR cases. This marker has been previously associated with acetaminophen- and phenytoin-induced hypersensitivity with small to moderate effect sizes (Jongkhajornpong et al., 2022; Tassaneeyakul et al., 2016). Among the 6 SCAR patients, three carried HLA-A*33:03, while two carried HLA-B*15:02. The high-risk alleles, HLA-B*57:01 and HLA-B*58:01, both strongly associated with drug-induced SCARs with large effect sizes, were detected in patients with SCARs and NIR-mild. Beta-lactam-associated alleles, including HLA-A*01:01, HLA-B*15:01, HLA-C*01:02, and HLA-C*06:02, were likewise detected in the cohort. In patients with a history of other drug allergy or co-allergies, HLA-A*33:03 was detected in 8 cases (40%), while HLA-B*58:01 and HLA-B*15:02 were detected in 5 (25%) and 3 (15%) cases, respectively (Figure 3).

### 3.3 Distribution of HLA alleles among cases and controls

All identified alleles in cases were sought in control populations as mentioned in the methodology. HLA alleles that had a frequency less than 0.01 were excluded from association analysis. This included HLA-DRB1*14:18, HLA-DQA1*01:05, HLA-DQA1*03:03, HLA-DPB1*14:02, and HLA-DPB1*100:01. The remaining alleles were subjected to association analysis with the control population, in which their allele frequencies of each dataset were demonstrated in Supplementary Figures 1, 2.

**FIGURE 4:**
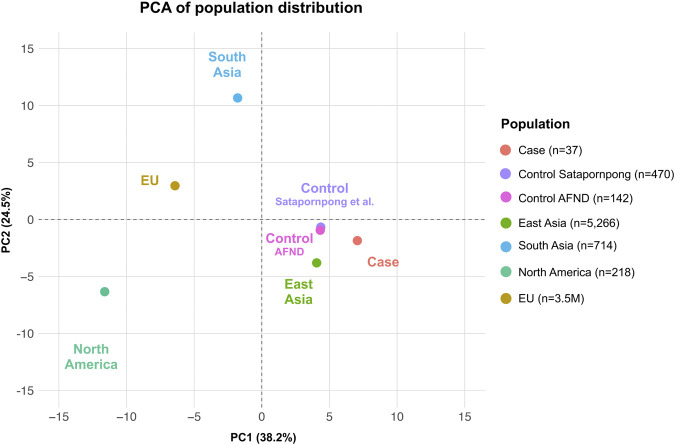
Population distribution of HLA alleles among case and control groups when compared with other population with different ethnicity. Allele frequency data of gold-standard populations from allele frequency net database (AFND) were extracted as follows: North America was from Mexico population, Europe (EU) from Germany and Kosovo, South Asia from Sri Lanka, and East Asia from Hong Kong population.

Population structure was analysed using AF from case (n = 37), control populations from Satapornpong et al. (n = 470) and AFND database of the Thailand population (n = 142), and four global populations from the gold-standard dataset of AFND. The HLA-DPB1 was then excluded from the analysis due to missing AF data, and the remaining 6 HLA genes were subjected to PCA. The first two principal components (PC1 and PC2) explained 38.2% and 24.5% of the total variance, respectively. The resulting PCA plot (Figure 4) illustrates the clustering pattern of the case and control populations relative to the global reference populations, reflecting genetic background and population structure based on allele frequencies of 6 HLA genes.

### 3.4 Candidate HLA allele signals in BL-HSR

**FIGURE 5:**
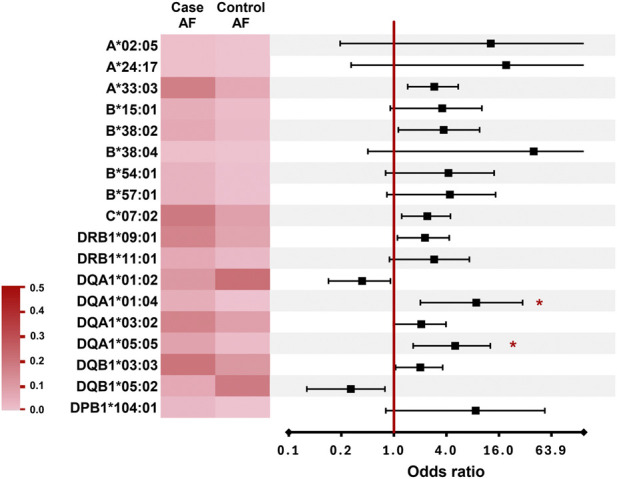
Association of HLA alleles with BL-HSR. AF, allele frequency; *significant after Bonferroni adjustment, color scale represents allele frequency.

Supplementary Tables 3–9 demonstrates association analysis for each HLA locus, and alleles that showed p-value less than 0.05 are illustrated in Figure 5. Among these, only HLA-DQA1*01:04 and HLA-DQA1*05:05 demonstrated potential association after Bonferroni correction, with odds ratio 8.79 (95% CI: 2.01–29.87; adjusted p-value: 0.029) and 5.03 (95% CI: 1.66–12.74; adjusted p-value: 0.029), respectively. Sensitivity analysis excluding rare alleles indicating that only HLA-A*33:03, HLA-C*07:02, HLA-DRB1*09:01, HLA-DQA1*03:02, HLA-DQB1*03:03 and HLA-DQB1*05:02 persisted with same effect sizes, while the top hits (HLA-DQA1*01:04, HLA-DQA1-05:05) were eliminated due to low allele frequency in control populations. Another sensitivity analysis using CADRs-free controls provided consistent results, although only 11 alleles were analyzed, and the other allele were excluded due to low frequency threshold in the control population (Supplementary Figure 3).

In addition, stratified analysis of HLA-A*33:03 according to co-allergy status suggested a relatively stronger signals among patients with co-allergies (OR 1.89, 95% CI 0.81–3.88), compared to those without co-allergies (OR 0.78, 95% CI 0.02–2.12); however, neither estimate reached statistical significance.

**FIGURE 6:**
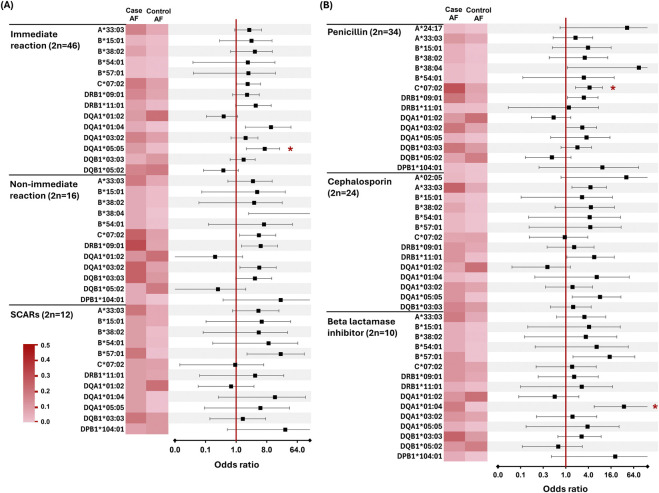
Subgroup analysis of association of HLA alleles and BL-HSR by **(A)** types of reaction and **(B)** culprit drugs. AF, allele frequency; SCARs, severe cutaneous adverse reactions; * significant after Bonferroni adjustment, color scale represents allele frequency.

Figure 6 illustrates subgroup analysis, and the results revealed consistent associations for types of reaction, where HLA-DQA1*05:05 appeared to show a similar direction of effect in IR and the effect size was slightly stronger, with OR 6.96 (95% CI: 2.01–19.25; adjusted p-value: 0.018). In subgroup analysis between culprit drugs, penicillin-induced reactions showed a prominent association in HLA-C*07:02, providing OR 4.31 (95% CI: 1.82–9.47; adjusted p-value: 0.009), and HLA-DQA1*01:04 in beta-lactamase inhibitor with OR 35.50 (95% CI: 5.69–153.47; adjusted p-value: 0.002).

Assessment of HLA haplotype profiles in Thai BL-HSR population was carried out. Among 37 samples, haplotype analysis found two to six combinations of HLA loci. The frequencies of each identified haplotype were reported in Supplementary Table 10. The association analysis was performed using haplotype AF reported in Geretz et al. (2018) as the control population and none of the those showed potential signal for BL-HSR.

### 3.5 Supporting analyses of HLA with BL-HSR using meta-analysis of association

To provide contextual comparison of evidence regarding candidate HLA variants and BL-HSR, a meta-analysis was performed using data from published studies with specified populations. Data from patients with BL-induced SCARs by Wattanachai et al. (2023) and patient**s** with cephalosporin-induced hypersensitivity by Wang et al. (Wang CC. et al., 2024) were included in this analysis. The pooled effect estimates for HLA alleles associated with SCARs demonstrated similar direction of effect for several alleles, including HLA-A*01:01, HLA-B*57:01, HLA-C*06:02, HLA-DRB1*04:05, HLA-DRB1*15:01, HLA-DQA1*03:01, and HLA-DQB1*03:02 (Supplementary Figure 4). However, meta-analysis of HLA alleles associated with cephalosporin allergy in Thai and Taiwan populations did not provide clear evidence of consistent pooled effects (Supplementary Figure 5).

## 4 Discussion

This study provided the first exploratory evidence of HLA class I and II associations with BL-HSR in Thai adult patients with confirmed allergy across both immediate, non-immediate and severe cutaneous adverse reactions. The study identified candidate susceptibility signals of two HLA class II alleles–HLA-DQA1*01:04 and HLA-DQA1*05:05 – among patients with confirmed BL-HSR. Subgroup analysis revealed enhanced signals of HLA-DQA1*05:05 in IR, while HLA-DQA1*01:04 were enriched in patients with allergy to beta-lactamase inhibitors. A potential effect for HLA-C*07:02 in penicillin-induced allergies was observed. Additionally, our meta-analyses provide contextual comparison with previously reported findings and highlight similarities and differences across populations.

Considerably, the pattern of HLA associations appears to vary markedly by population and phenotype. In a large genome-wide association study from United Kingdom (UK), HLA-B*55:01 was identified as a risk allele for penicillin allergy in a predominantly European population (Krebs et al., 2020), while a study of deeply phenotyped immediate BL reactions from Australia, France, Spain, Italy, and UK identified HLA-DRB1*10:01 as risk factor even without HLA-DQA1*01:05 allele (Nicoletti et al., 2021). Another study in Italian population found a strong association between HLA-DRB3*02:02 and delayed penicillin hypersensitivity when comparing with immediate-type reaction (Romano et al., 2022). These findings suggest that both HLA class I and class II may contribute to BL-HSR, although the specific alleles involved may differ across populations, even within European population. Meanwhile, in Asian populations, several studies exhibit distinct signals. Taiwanese patients demonstrated associations with HLA-DPB1*05:01 and HLA-B*55:02 (Wang CC. et al., 2024), while a Han Chinese study found that HLA-B*15:01 was a potential key risk factor for amoxicillin-induced SCAR (Wang T. et al., 2024). In our study, we found candidate signals in HLA-DQA1*01:04 and HLA-DQA1*05:05. Though the allele were not specifically similar to the study in UK, HLA-DQA1 support evidence of being a potential predisposing factor to BL-HSH. However, such differences may reflect underlying population‐specific allele frequencies and differences in genetic backgrounds.

In addition, there appears to be considerable heterogeneity even in Thai population. Our findings contrast with prior Thai pediatric study, highlighting potential differences between pediatric and adult cohorts. HLA-B*48:01 and HLA-C*04:06 were strong risk factors in Thai children (Singvijarn et al., 2021) but were absent in our adult patient group, despite having been used to inform our sample size calculation. This discrepancy suggests that age-related differences may influence the clinical phenotype of BL-HSR and, in turn, contribute to differences in HLA allele distribution across age groups. Furthermore, a prior study on BL-induced SCARs in Thai adults reported a tendency towards association with a different set of alleles (e.g., HLA-A*01:01, HLA-B*50:01) (Wattanachai et al., 2023), which were observed at low frequencies in our overall cohort. This cumulative evidence might suggest a complex, multifactorial genetic architecture for BL hypersensitivity, where genetic risk is heavily influenced by age, phenotype reaction, and culprit drug.

To contextualize our primary findings in regional context, we performed a meta-analysis incorporating published data on BL-HSR across Thai and Asian populations (Wang CC. et al., 2024; Wattanachai et al., 2023). Specifically, the pooled effect estimates for SCARs demonstrated consistent direction of effect for several high-risk alleles, including HLA-A*01:01 and HLA-B*57:01, supporting our findings as potential markers in SCARs, but warrant further validation. However, the analysis of cephalosporin allergy with Taiwanese data showed high heterogeneity and inverse effects, suggesting that HLA-mediated risk is heterogeneous and dependent on genetic background (Lumk et al., 2023). For other SCAR markers, though our study includes patients with SCARs and those well-recognized alleles (i.e., HLA-B*15:02, HLA-B*58:01) were observed, their signals were not dominant in our analysis. HLA-B*15:02 is a well-established risk allele for carbamazepine-induced SJS/TEN in Southeast Asian populations; however, its role in beta-lactam hypersensitivity might be less specific, suggesting that immunopathogenic mechanisms underlying antiepileptic–induced SCARs may differ substantially from those of beta-lactam–induced reactions, which are typically hapten-mediated.

Furthermore, we noted that more than half of our patients reported allergies to other, unrelated drugs such as fluoroquinolones, sulfonamides, and NSAIDs. This high rate of co-occurring drug allergies may suggest the presence of MDH syndrome in a subset of patients, which is characterized by a long-lasting T-cell hyperresponsiveness to different, structurally unrelated drugs and mostly present**s** with severe exanthems or DRESS as an initial symptom (Pichler et al., 2017). Also, MDH has been known to be associated with family history (Kurtz et al., 2000). However, none of our severe cases reported other drug allergy history. In non-severe cases, we found that HLA-A*33:03 was detected in 40% of patients with co-allergies compared with 18% in those without, though the stratified analysis did not suggest candidate susceptibility signals. Prior reports have demonstrated an association between HLA-A*33:03 and to acetaminophen- and phenytoin-induced hypersensitivity (Jongkhajornpong et al., 2022; Tassaneeyakul et al., 2016), suggesting that certain HLA alleles might predispose individuals to a broader immunologic hyper-reactivity, possibly accounting for the elevated risk of multiple drug allergies (Pichler et al., 2017). However, we acknowledge that multiple reported allergies could also stem from mislabeling or shared risk factors in atopy, which requires further study.

Mechanistically, the identification of class II alleles may suggest a potential role of CD4^+^ T cells in BL hypersensitivity. However, given the exploratory nature of this study, no definitive causal conclusion should be drawn. Indeed, BL antibiotics are well-known haptens that covalently bind to host proteins to form drug–protein conjugates (Levine and Ovary, 1961). Even in typically IgE-mediated IR, HLA Class II molecules may be vital for presenting BL-derived peptides to T helper cells, thereby driving a robust Th2 response necessary for IgE class-switching (Shenoy et al., 2019; Ariza et al., 2015). Variability in the peptide-binding groove of specific HLA alleles may influence the affinity and stability of drug-modified peptide presentation. The enhanced effects observed of HLA-DQA1*05:05 with IR may suggest that this specific allele is possibly efficient at presenting the drug-derived peptides. Alternatively, the response could be explained by the pharmacologic interaction (p-i) hypothesis (Pichler, 2002), where BL derivatives directly bind to immune receptors without conventional processing. It is plausible that the unique peptide-binding motifs of such alleles either favor the presentation of BL hapten epitopes or facilitate a p-i mode of T-cell stimulation, thus escalating the immune response. Although these mechanisms remain speculative in our cohort, these hypotheses are biologically plausible and consistent with current models of drug hypersensitivity.

For clinical perspective, our findings are not sufficient to support clinical screening or individual risk prediction and the gold standard for BL-HSR confirmation remains a thorough work-up involving skin testing and drug provocation tests (Shenoy et al., 2019). The findings raise the possibility that patients with a history of severe drug reactions, including SCARs, may harbor SCAR-related HLA signals, particularly in the context of MDH, providing baseline evidence for guiding future studies.

### 4.1 Strength, limitations and future directions

To the best of our knowledge, this study is one of the first exploratory report in Thailand to examine HLA profile across the spectrum of BL-HSR phenotypes in adults, including IR, NIR-mild, and SCARs. All of our patients were confirmed using drug provocation or skin test except in severe cases. We used 4-digit-resolution sequencing-based HLA typing, which improves accuracy in allele calling compared with serological or low-resolution methods. We also leveraged a large aggregate control dataset from multiple sources, which provided a reasonable approximation of allele frequencies in the background population.

However, several limitations should be emphasized. First, the sample size was relatively small. While our sample size justification was based on anticipated allele frequencies, the multiple comparisons had a considerable risk of both false negatives and false positives. This risk was further enhanced by phenotype stratification, which significantly limited our statistical power. The effect size of HLA-DQA1 alleles could be overestimated due to a small discovery sample.

Second, the use of pooled control allele frequencies instead of direct genotyping of controls may introduce bias and limits the ability to infer individual-level risk. We assumed that the general Thai population allele frequency is a valid comparator, but subtle population stratification could somehow bias the results. We tried to mitigate this by performing a principal component analysis to ensure our cases cluster with the reference Thai populations, and this showed no major outliers in our sample structure. Moreover, our study did not include solely BL tolerant controls which hinder significant interpretation of the results. However, a substantial proportion of our reference dataset was derived from a nationwide Thai cohort in which control individuals were defined as healthy subjects with no history of CADRs. Though our sensitivity analysis among these control subjects revealed consistent effects, conclusion should not be made and future studies incorporating matched BL tolerant controls are necessary to determine predictive values and clinical utility. Moreover, the observed associations were not robust in sensitivity analyses, where primary signals were attenuated after restricting to more frequent alleles, suggesting that the findings may be sensitive to analytical assumptions.

Third, the heterogeneity of our patient cases could also be considered a limitation since we combined different reaction types under the umbrella of BL hypersensitivity. This was by design, to search for common genetic denominators, but it likely dilutes associations specific to a given phenotype. Our subgroup analysis partly addressed this gap, though interpreting the effect size should be done with caution.

Fourth, due to strong linkage disequilibrium across HLA loci, unraveling independent genetic effects from correlated signals is difficult. While haplotype analysis was performed, our design does not allow us to determine whether observed signals reflect independent allele effects or are secondary to a correlated haplotypic background.

Therefore, there is a clear need to replicate and expand these results in a larger prospective nationwide study or an Asian consortium study. Such studies should ideally include phenotype stratification (separating IR from NIR, and/or mild from severe reactions) to accurately detect phenotype-specific HLA effects. Furthermore, given the multiple drug allergy tendency observed, future research could investigate whether certain HLA alleles predispose individuals to multiple different drug allergies or an overactive immune response. Also, mechanistic and functional experiments including *in silico* structural modeling of beta-lactam binding to DQA1 molecules, *in vitro* peptide-binding or elution assays specifically for these alleles, and drug-specific T-cell proliferation or cytokine-release assays in allele carriers are warranted. These experiments would help determine whether the observed signals represent potential link to immunological activation and provide robust evidence for the association between HLA structures and immune response.

## 5 Conclusion

In this multi-center study of Thai adult patients with BL-HSR, we described clinical patterns of BL-HSR and identified candidate HLA class II susceptibility signals. HLA-DQA1*01:04 and HLA-DQA1*05:05 may be associated with BL-HSR; HLA-DQA1*05:05 showed stronger signal in patients with immediate reactions, while HLA-DQA1*01:04 was enriched in patients responded to β-lactamase inhibitors. Meta-analysis provided contextual results among Thai patients with SCARs and Asian populations revealing genetically distinct patterns. Our findings should be interpreted as exploratory and hypothesis-generating, providing preliminary insights into population-specific genetic signals to support future studies on the immunogenetic risk stratification of BL-HSR.

## Data Availability

The HLA allele frequency summary data and association statistics generated in this study are provided in the Supplementary Material and/or deposited in the Allele Frequency Net Database (AFND), where applicable. De-identified individual-level sequencing data may be made available from the corresponding author upon reasonable request, subject to approval by the relevant institutional ethics committee and completion of an appropriate data access agreement.

## References

[B1] Ariza A. Mayorga C. Fernandez T. D. Barbero N. Martín-Serrano A. Pérez-Sala D. (2015). Hypersensitivity reactions to β-lactams: relevance of hapten-protein conjugates. J. Investig. Allergol. Clin. Immunol. 25 (1), 12–25. 25898690

[B2] Barquera R. Zuniga J. Flores-Rivera J. Corona T. Penman B. S. Hernández-Zaragoza D. I. (2020). Diversity of HLA class I and class II blocks and conserved extended haplotypes in lacandon mayans. Sci. Rep. 10 (1), 3248. 10.1038/s41598-020-58897-5 32094421 PMC7039995

[B3] Brockow K. Ardern-Jones M. R. Mockenhaupt M. Aberer W. Barbaud A. Caubet J. C. (2019). EAACI position paper on how to classify cutaneous manifestations of drug hypersensitivity. Allergy 74 (1), 14–27. 10.1111/all.13562 30028512

[B4] Calle A. M. Aguirre N. Ardila J. C. Cardona Villa R. (2023). DRESS syndrome: a literature review and treatment algorithm. World Allergy Organ J. 16 (3), 100673. 10.1016/j.waojou.2022.100673 37082745 PMC10112187

[B5] Chandanayingyong D. Stephens H. A. Fan L. Sirikong M. Longta P. Vangseratthana R. (1994). HLA-DPB1 polymorphism in the thais of southeast Asia. Hum. Immunol. 40 (1), 20–24. 10.1016/0198-8859(94)90017-5 8045789

[B6] Chandanayingyong D. Stephens H. A. Klaythong R. Sirikong M. Udee S. Longta P. (1997). HLA-A, -B, -DRB1, -DQA1, and -DQB1 polymorphism in thais. Hum. Immunol. 53 (2), 174–182. 10.1016/S0198-8859(96)00284-4 9129976

[B7] Chongpison Y. Palapinyo S. Mongkolpathumrat P. Buranapraditkun S. Thantiworasit P. Klaewsongkram J. (2023). Beta-lactam hypersensitivity diagnosis in ambulatory and hospitalized settings require different approaches. Ann. Allergy Asthma Immunol. 130 (1), 84–92.e1. 10.1016/j.anai.2022.09.011 36122888

[B8] Gerberick G. F. Troutman J. A. Foertsch L. M. Vassallo J. D. Quijano M. Dobson R. L. (2009). Investigation of peptide reactivity of pro-hapten skin sensitizers using a peroxidase-peroxide oxidation system. Toxicol. Sci. 112 (1), 164–174. 10.1093/toxsci/kfp192 19748994

[B9] Geretz A. Ehrenberg P. K. Bouckenooghe A. Fernández Viña M. A. Michael N. L. Chansinghakule D. (2018). Full-length next-generation sequencing of HLA class I and II genes in a cohort from Thailand. Hum. Immunol. 79 (11), 773–780. 10.1016/j.humimm.2018.09.005 30243890

[B10] Gonzalez-Galarza F. F. McCabe A. Santos E. Jones J. Takeshita L. Ortega-Rivera N. D. (2020). Allele frequency net database (AFND) 2020 update: gold-standard data classification, open access genotype data and new query tools. Nucleic Acids Res. 48 (D1), D783. 10.1093/nar/gkz1029 31722398 PMC7145554

[B11] Grifoni A. Weiskopf D. Lindestam Arlehamn C. S. Angelo M. Leary S. Sidney J. (2018). Sequence-based HLA-A, B, C, DP, DQ, and DR typing of 714 adults from Colombo, Sri Lanka. Hum. Immunol. 79 (2), 87–88. 10.1016/j.humimm.2017.12.007 29289740 PMC6378951

[B12] Hsu D. Y. Brieva J. Silverberg N. B. Silverberg J. I. (2016). Morbidity and mortality of Stevens-Johnson syndrome and toxic epidermal necrolysis in United States adults. J. Invest. Dermatol 136 (7), 1387–1397. 10.1016/j.jid.2016.03.023 27039263

[B13] Jeimy S. Ben-Shoshan M. Abrams E. M. Ellis A. K. Connors L. Wong T. (2020). Practical guide for evaluation and management of beta-lactam allergy: position statement from the Canadian society of allergy and clinical immunology. Allergy Asthma Clin. Immunol. 16 (1), 95. 10.1186/s13223-020-00494-2 33292466 PMC7653726

[B14] Jongkhajornpong P. Ueta M. Lekhanont K. Puangsricharern V. Prabhasawat P. Chantaren P. (2022). Association of HLA polymorphisms and acetaminophen-related steven-johnson syndrome with severe ocular complications in Thai population. Br. J. Ophthalmol. 106 (6), 884–888. 10.1136/bjophthalmol-2020-317315 33229345

[B15] Kawaguchi S. Matsuda F. (2020). High-definition genomic analysis of HLA genes *via* comprehensive HLA allele genotyping. Methods Mol. Biol. 2131, 31–38. 10.1007/978-1-0716-0389-5_3 32162249

[B16] Kawaguchi S. Higasa K. Shimizu M. Yamada R. Matsuda F. (2017). HLA-HD: an accurate HLA typing algorithm for next-generation sequencing data. Hum. Mutat. 38 (7), 788–797. 10.1002/humu.23230 28419628

[B17] Kawaguchi S. Higasa K. Yamada R. Matsuda F. (2018). Comprehensive HLA typing from a current allele database using next-generation sequencing data. Methods Mol. Biol. 1802, 225–233. 10.1007/978-1-4939-8546-3_16 29858813

[B18] Kloypan C. Koomdee N. Satapornpong P. Tempark T. Biswas M. Sukasem C. (2021). A comprehensive review of HLA and severe cutaneous adverse drug reactions: implication for clinical pharmacogenomics and precision medicine. Pharm. (Basel) 14 (11), 1077. 10.3390/ph14111077 34832859 PMC8622011

[B19] Krebs K. Bovijn J. Zheng N. Lepamets M. Censin J. C. Jürgenson T. (2020). Genome-wide study identifies association between HLA-B(∗)55:01 and self-reported penicillin allergy. Am. J. Hum. Genet. 107 (4), 612–621. 10.1016/j.ajhg.2020.08.008 32888428 PMC7536643

[B20] Kurtz K. M. Beatty T. L. Adkinson N. F. Jr (2000). Evidence for familial aggregation of immunologic drug reactions. J. Allergy Clin. Immunol. 105 (1 Pt 1), 184–185. 10.1016/s0091-6749(00)90196-9 10629471

[B21] Kwok J. Tang W. H. Chu W. K. Chan Y. S. Liu Z. Yang W. (2020). High resolution allele genotyping and haplotype frequencies for NGS based HLA 11 loci of 5266 Hong Kong Chinese bone marrow donors. Hum. Immunol. 81 (10-11), 577–579. 10.1016/j.humimm.2020.08.005 32893027

[B22] Levine B. B. Ovary Z. (1961). Studies on the mechanism of the formation of the penicillin antigen. III. The N-(D-alpha-benzylpenicilloyl) group as an antigenic determinant responsible for hypersensitivity to penicillin G. J. Exp. Med. 114 (6), 875–904. 10.1084/jem.114.6.875 14464604 PMC2180410

[B23] Li K. Lauschke V. M. Zhou Y. (2025). Molecular docking to investigate HLA-associated idiosyncratic drug reactions. Drug Metab. Rev. 57 (1), 67–90. 10.1080/03602532.2025.2453521 39811883

[B24] Lumkul L. Wongyikul P. Kulalert P. Sompornrattanaphan M. Lao-Araya M. Chuamanochan M. (2023). Genetic association of beta-lactams-induced hypersensitivity reactions: a systematic review of genome-wide evidence and meta-analysis of candidate genes. World Allergy Organ J. 16 (9), 100816. 10.1016/j.waojou.2023.100816 37780578 PMC10541471

[B25] Maneemaroj R. Stephens H. A. Chandanayingyong D. Longta K. Bejrachandra S. (1997). HLA class II allele frequencies in northern thais (kamphaeng phet). J. Med. Assoc. Thai 80 (Suppl. 1), S20–S24. 9347641

[B26] Martin M. A. Hoffman J. M. Freimuth R. R. Klein T. E. Dong B. J. Pirmohamed M. (2014). Clinical pharmacogenetics implementation consortium guidelines for HLA-B genotype and abacavir dosing: 2014 update. Clin. Pharmacol. Ther. 95 (5), 499–500. 10.1038/clpt.2014.38 24561393 PMC3994233

[B27] Matzinger P. (1994). Tolerance, danger, and the extended family. Annu. Rev. Immunol. 12, 991–1045. 10.1146/annurev.iy.12.040194.005015 8011301

[B28] Micheletti R. G. Chiesa-Fuxench Z. Noe M. H. Stephen S. Aleshin M. Agarwal A. (2018). Stevens-johnson syndrome/toxic epidermal necrolysis: a multicenter retrospective study of 377 adult patients from the United States. J. Invest. Dermatol 138 (11), 2315–2321. 10.1016/j.jid.2018.04.027 29758282

[B29] Nathalang O. Tatsumi N. Hino M. Prayoonwiwat W. Yamane T. Suwanasophon C. (1999). HLA class II polymorphism in Thai patients with non-hodgkin's lymphoma. Eur. J. Immunogenet 26 (6), 389–392. 10.1046/j.1365-2370.1999.00177.x 10583459

[B30] Nicoletti P. Carr D. F. Barrett S. McEvoy L. Friedmann P. S. Shear N. H. (2021). Beta-lactam-induced immediate hypersensitivity reactions: a genome-wide association study of a deeply phenotyped cohort. J. Allergy Clin. Immunol. 147 (5), 1830–1837.e15. 10.1016/j.jaci.2020.10.004 33058932 PMC8100096

[B31] Parisi R. Shah H. Navarini A. A. Muehleisen B. Ziv M. Shear N. H. (2023). Acute generalized exanthematous pustulosis: clinical features, differential diagnosis, and management. Am. J. Clin. Dermatol 24 (4), 557–575. 10.1007/s40257-023-00779-3 37156992 PMC10166469

[B32] Phillips E. J. Sukasem C. Whirl-Carrillo M. Müller D. J. Dunnenberger H. M. Chantratita W. (2018). Clinical pharmacogenetics implementation consortium guideline for HLA genotype and use of carbamazepine and oxcarbazepine: 2017 update. Clin. Pharmacol. Ther. 103 (4), 574–581. 10.1002/cpt.1004 29392710 PMC5847474

[B33] Pichler W. J. (2002). Pharmacological interaction of drugs with antigen-specific immune receptors: the p-i concept. Curr. Opin. Allergy Clin. Immunol. 2 (4), 301–305. 10.1097/00130832-200208000-00003 12130944

[B34] Pichler W. J. Srinoulprasert Y. Yun J. Hausmann O. (2017). Multiple drug hypersensitivity. Int. Arch. Allergy Immunol. 172 (3), 129–138. 10.1159/000458725 28315874 PMC5472211

[B35] Pimtanothai N. Charoenwongse P. Mutirangura A. Hurley C. K. (2002). Distribution of HLA-B alleles in nasopharyngeal carcinoma patients and normal controls in Thailand. Tissue Antigens 59 (3), 223–225. 10.1034/j.1399-0039.2002.590308.x 12074714

[B36] Romano A. Oussalah A. Chery C. Guéant-Rodriguez R. M. Gaeta F. Cornejo-Garcia J. A. (2022). Next-generation sequencing and genotype association studies reveal the association of HLA-DRB3*02:02 with delayed hypersensitivity to penicillins. Allergy 77 (6), 1827–1834. 10.1111/all.15147 34687232

[B37] Romphruk A. V. Romphruk A. Kongmaroeng C. Klumkrathok K. Paupairoj C. Leelayuwat C. (2010). HLA class I and II alleles and haplotypes in ethnic northeast thais. Tissue Antigens 75 (6), 701–711. 10.1111/j.1399-0039.2010.01448.x 20230525

[B38] Sangasapasviliya A. Prakongwong T. Ayuthaya P. K. Ayuthaya R. K. N. (2010). Drug hypersensitivity in phramongkutklao hospital. J. Med. Assoc. Thai 93 (Suppl. 6), S106–S111. 21280522

[B39] Satapornpong P. Jinda P. Jantararoungtong T. Koomdee N. Chaichan C. Pratoomwun J. (2020). Genetic diversity of HLA class I and class II alleles in Thai populations: contribution to genotype-guided therapeutics. Front. Pharmacol. 11, 78. 10.3389/fphar.2020.00078 32180714 PMC7057685

[B40] Schaid D. J. Rowland C. M. Tines D. E. Jacobson R. M. Poland G. A. (2002). Stats: statistical analysis of haplotypes with traits and covariates when linkage phase is ambiguous. 1.9.7 ed2024. 10.1086/338688 PMC384917 11791212

[B41] Seitz S. Lange V. Norman P. J. Sauter J. Schmidt A. H. (2021). Estimating HLA haplotype frequencies from homozygous individuals - a technical report. Int. J. Immunogenet 48 (6), 490–495. 10.1111/iji.12553 34570965 PMC9131737

[B42] Shenoy E. S. Macy E. Rowe T. Blumenthal K. G. (2019). Evaluation and management of penicillin allergy: a review. Jama 321 (2), 188–199. 10.1001/jama.2018.19283 30644987

[B43] Singvijarn P. Manuyakorn W. Mahasirimongkol S. Wattanapokayakit S. Inunchot W. Wichukchinda N. (2021). Association of HLA genotypes with beta-lactam antibiotic hypersensitivity in children. Asian Pac J. Allergy Immunol. 39 (3), 197–205. 10.12932/AP-271118-0449 31012593

[B44] Sirikong M. Tsuchiya N. Chandanayingyong D. Bejrachandra S. Suthipinittharm P. Luangtrakool K. (2002). Association of HLA-DRB1*1502-DQB1*0501 haplotype with susceptibility to systemic lupus erythematosus in thais. Tissue Antigens 59 (2), 113–117. 10.1034/j.1399-0039.2002.590206.x 12028537

[B45] Sousa-Pinto B. Fonseca J. A. Gomes E. R. (2017). Frequency of self-reported drug allergy: a systematic review and meta-analysis with meta-regression. Ann. Allergy Asthma Immunol. 119 (4), 362–373.e2. 10.1016/j.anai.2017.07.009 28779998

[B46] Tantikul C. Dhana N. Jongjarearnprasert K. Visitsunthorn N. Vichyanond P. Jirapongsananuruk O. (2008). The utility of the world health organization-the Uppsala monitoring centre (WHO-UMC) system for the assessment of adverse drug reactions in hospitalized children. Asian Pac J. Allergy Immunol. 26 (2-3), 77–82. 19054924

[B47] Tassaneeyakul W. Prabmeechai N. Sukasem C. Kongpan T. Konyoung P. Chumworathayi P. (2016). Associations between HLA class I and cytochrome P450 2C9 genetic polymorphisms and phenytoin-related severe cutaneous adverse reactions in a Thai population. Pharmacogenet Genomics. 26 (5), 225–234. 10.1097/FPC.0000000000000211 26928377

[B48] Thailand (2002). Allele Frequency Net Database. Available online at: https://www.allelefrequencies.net/pop6001c.asp?pop_name=Thailand (Accessed December 23, 2025).

[B49] Thakuria B. Lahon K. (2013). The beta lactam antibiotics as an empirical therapy in a developing country: an update on their current status and recommendations to counter the resistance against them. J. Clin. Diagn Res. 7 (6), 1207–1214. 10.7860/JCDR/2013/5239.3052 23905143 PMC3708238

[B50] TusfaD A. (2016). Table of Pharmacogenomic Biomarkers in Drug Labeling. *U.S. Food and Drug Administration* . Available online at: https://www.fda.gov/drugs/science-and-research-drugs/table-pharmacogenomic-biomarkers-drug-labeling (Access June 16, 2026).

[B51] Wang C. C. Shen C. H. Lin G. C. Chen Y. M. Chen I. C. (2024). Association of HLA alleles with cephalosporin allergy in the Taiwanese population. Sci. Rep. 14 (1), 17167. 10.1038/s41598-024-68185-1 39060355 PMC11282083

[B52] Wang T. Yang J. Yang F. Cheng Y. Huang Z. Li B. (2024). The association between HLA-B variants and amoxicillin-induced severe cutaneous adverse reactions in Chinese han population. Front. Pharmacol. 15, 1400239. 10.3389/fphar.2024.1400239 38863977 PMC11165025

[B53] Wattanachai P. Amornpinyo W. Konyoung P. Purimart D. Khunarkornsiri U. Pattanacheewapull O. (2023). Association between HLA alleles and beta-lactam antibiotics-related severe cutaneous adverse reactions. Front. Pharmacol. 14, 1248386. 10.3389/fphar.2023.1248386 37795024 PMC10546186

[B54] Wongsurawat T. Nakkuntod J. Charoenwongse P. Snabboon T. Sridama V. Hirankarn N. (2006). The association between HLA class II haplotype with graves' disease in Thai population. Tissue Antigens 67 (1), 79–83. 10.1111/j.1399-0039.2005.00498.x 16451208

[B55] Zhou L. Dhopeshwarkar N. Blumenthal K. G. Goss F. Topaz M. Slight S. P. (2016). Drug allergies documented in electronic health records of a large healthcare system. Allergy 71 (9), 1305–1313. 10.1111/all.12881 26970431 PMC12841114

[B56] Zhou Y. Krebs K. Milani L. Lauschke V. M. (2021). Global frequencies of clinically important HLA alleles and their implications for the cost-effectiveness of preemptive pharmacogenetic testing. Clin. Pharmacol. Ther. 109 (1), 160–174. 10.1002/cpt.1944 32535895

