# Supplementary material for: HLA allele profiling and association in Thai patients with confirmed beta-lactam hypersensitivity: an NGS-based analysis
Source: Front Pharmacol. 2026 Jul 10;17:1782094. doi: 10.3389/fphar.2026.1782094 (PMC13395944; doi:10.3389/fphar.2026.1782094)
Supplement: Supplementary file 1 [file Supplementaryfile1.docx]

Supplementary Material

**Supplementary Figure 1** Allele frequency of HLA types for HLA-A, HLA-B, HLA-C, HLA-DQA1 found in general Thai population. AFND, Allele frequency net database

**Supplementary Figure 2** Allele frequency of HLA types for HLA-DRB1, HLA-DQB1 and HLA-DPB1 found in general Thai population

**Supplementary Figure 3** Sensitivity analysis for association of HLA alleles with non-adjust significant, when control data with no cutaneous adverse reaction were included (n=470). Results were compared with primary analysis with odds ration and 95% confidence interval (95%CI).

**Supplementary Figure 4** Meta-analysis of HLA associations in patients with SCARs

**Supplementary Figure 5** Meta-analysis of HLA associations in patients with cephalosporin allergy

**Supplementary Table 1** HLA class I genotypes of 37 BL-HSR cases

**Supplementary Table 2** HLA class II genotypes of 37 BL-HSR cases

**Supplementary Table 3** Association analysis of HLA-A alleles and BL-HSR

**Supplementary Table 4** Association analysis of HLA-B alleles and BL-HSR

**Supplementary Table 5** Association analysis of HLA-C alleles and BL-HSR

**Supplementary Table 6** Association analysis of HLA-DRB1 alleles and BL-HSR

**Supplementary Table 7** Association analysis of HLA-DQA1 alleles and BL-HSR

**Supplementary Table 8** Association analysis of HLA-DQB1 alleles and BL-HSR

**Supplementary Table 9** Association analysis of HLA-DPB1 alleles and BL-HSR

**Supplementary Table 10** Association of HLA haplotypes in patients with BL-HSR reactions and Thai population

**
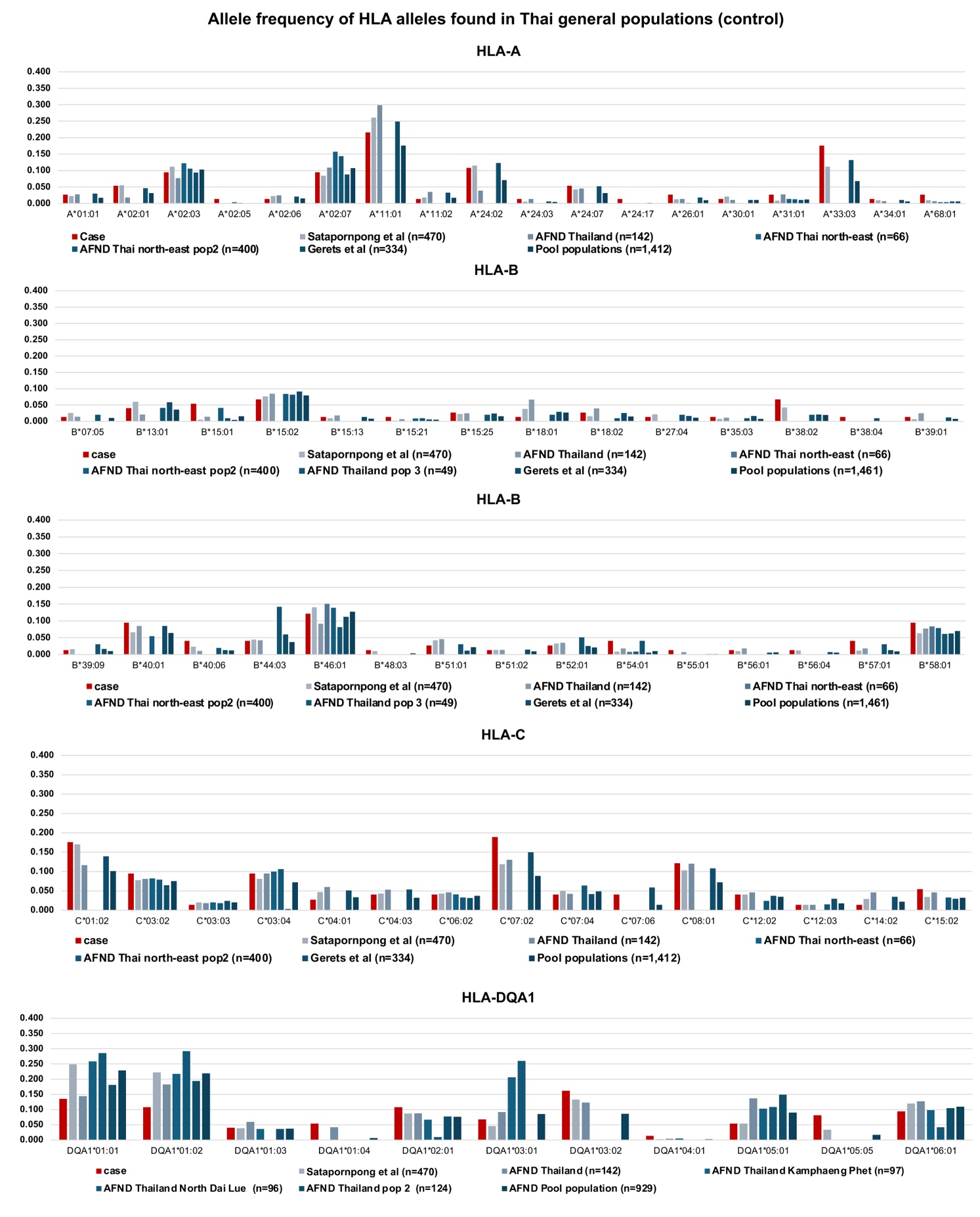
Supplementary Figure 1.** Allele frequency of HLA types for HLA-A, HLA-B, HLA-C, HLA-DQA1 found in general Thai population. AFND, Allele frequency net database.

**
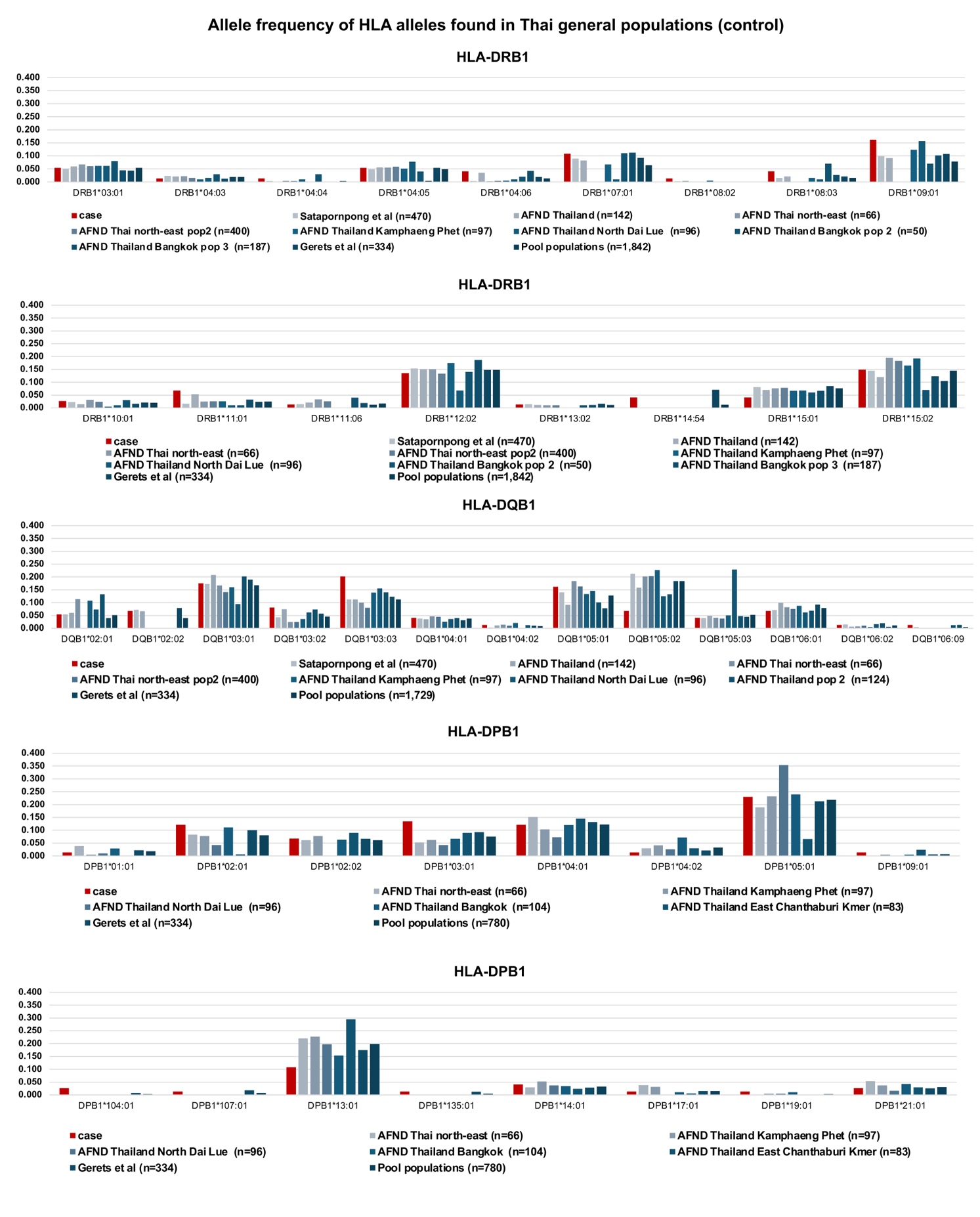
Supplementary Figure 2.** Allele frequency of HLA types for HLA-DRB1, HLA-DQB1 and HLA-DPB1 found in general Thai population. AFND, Allele frequency net database.

**Supplementary Figure 3** Sensitivity analysis for association of HLA alleles with non-adjust significant, when control data with no cutaneous adverse reaction were included (n=470). Results were compared with primary analysis with odds ration and 95% confidence interval (95%CI).

**
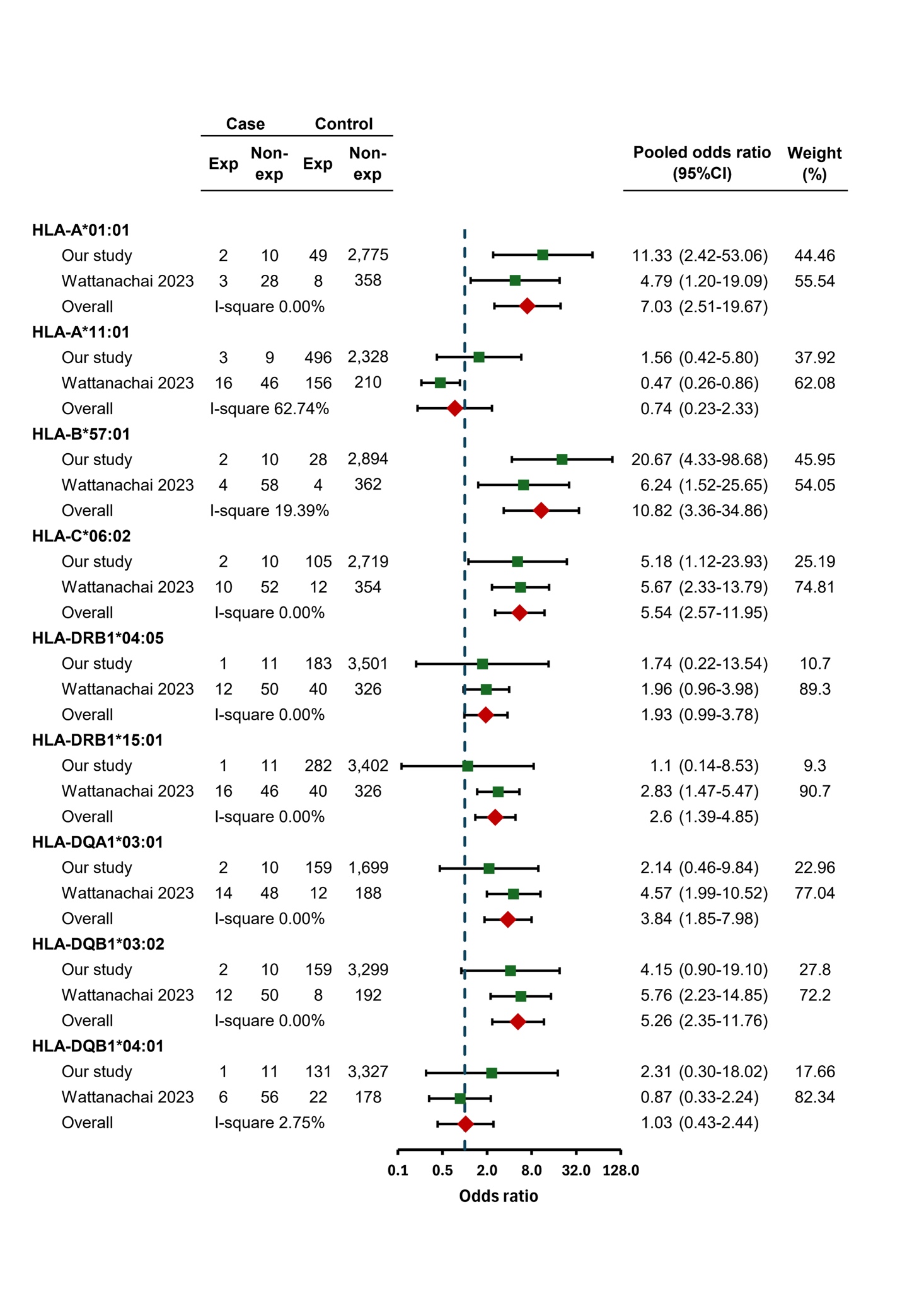
**

**Supplementary Figure 4** Meta-analysis of HLA associations in patients with SCARs

**
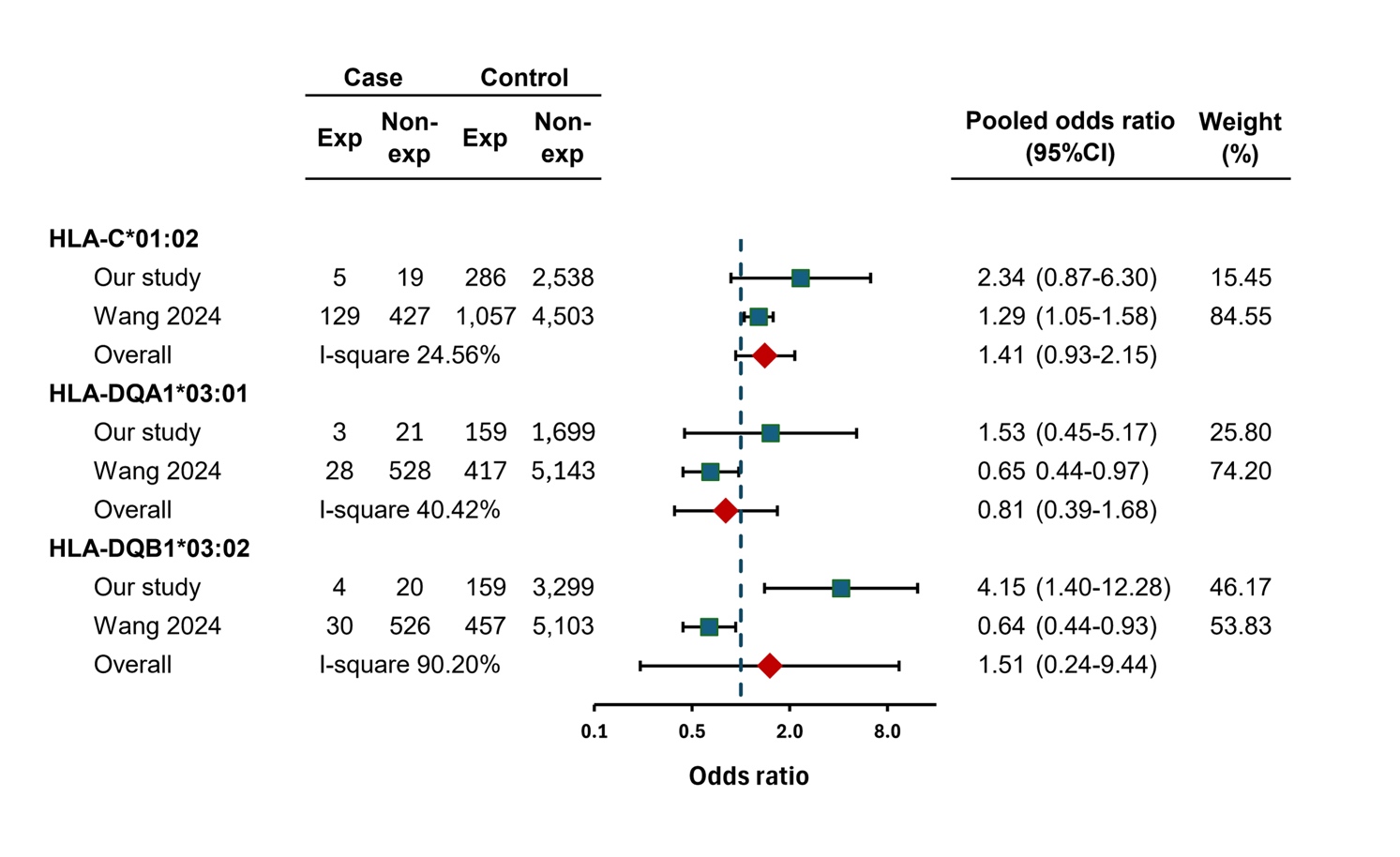
**

**Supplementary Figure 5** Meta-analysis of HLA associations in patients with cephalosporin allergy

**Supplementary Table 1** HLA class I genotypes of 37 BL-HSR cases

| **ID** | **Reaction Type** | **Clinical** | **Drug** | **Confirm test** | **HLA-A** | | **HLA-B** | | **HLA-C** | |
| --- | --- | --- | --- | --- | --- | --- | --- | --- | --- | --- |
| 1 | IR | Urticaria | Penicillin | SPT, DPT | A*30:01 | A*68:01 | B*07:05 | B*56:01 | C*07:02 | C*12:02 |
| 2 | IR | Angioedema | Amoxicillin | SPT, DPT | A*11:01 | A*24:02 | B*13:01 | B*40:01 | C*03:04 | C*03:04 |
| 3 | IR | Anaphylaxis | Penicillin | SPT | A*02:07 | A*33:03 | B*46:01 | B*51:01 | C*01:02 | C*15:02 |
| 4 | IR | Angioedema | Ampicillin, Amoxicillin | SPT | A*24:02 | A*34:01 | B*15:21 | B*18:02 | C*04:03 | C*07:04 |
| 5 | NIR-mild | Fixed drug eruption | Penicillin | IDT | A*02:07 | A*11:01 | B*40:01 | B*46:01 | C*01:02 | C*07:02 |
| 6 | NIR-mild | MPE | Penicillin | SPT, DPT | A*33:03 | A*33:03 | B*44:03 | B*40:06 | C*07:06 | C*15:02 |
| 7 | NIR-mild | Angioedema, MPE | Amoxicillin, Penicillin | SPT, DPT | A*24:02 | A*31:01 | B*40:01 | B*40:06 | C*03:04 | C*08:01 |
| 8 | NIR-mild | MPE | Ampicillin, Penicillin | SPT,IDT | A*11:01 | A*33:03 | B*38:02 | B*56:04 | C*01:02 | C*07:02 |
| 9 | IR | Anaphylaxis | Amoxicillin | SPT,IDT,DPT | A*02:03 | A*24:02 | B*18:02 | B*18:01 | C*07:04 | C*07:04 |
| 10 | IR | Angioedema, urticaria | Penicillin | SPT,IDT | A*02:07 | A*24:02 | B*46:01 | B*54:01 | C*01:02 | C*03:04 |
| 11 | IR | Urticaria | Cloxacillin | SPT,IDT | A*24:17 | A*31:01 | B*39:01 | B*51:02 | C*07:02 | C*15:02 |
| 12 | NIR-mild | Angioedema | Augmentin | SPT,IDT,DPT | A*02:01 | A*02:03 | B*40:01 | B*54:01 | C*01:02 | C*03:03 |
| 13 | NIR-mild | Angioedema | Dicloxacillin | SPT,IDT,DPT | A*02:01 | A*02:07 | B*15:01 | B*15:02 | C*07:02 | C*08:01 |
| 14 | NIR-mild | MPE | Penicillin | SPT,IDT,DPT | A*11:01 | A*26:01 | B*15:13 | B*55:01 | C*01:02 | C*08:01 |
| 15 | IR | Urticaria | Cephalexin | SPT,IDT,DPT | A*02:07 | A*11:01 | B*46:01 | B*51:01 | C*01:02 | C*14:02 |
| 16 | SCARs | AGEP | Cefazolin | Clinical | A*11:01 | A*33:03 | B*35:03 | B*54:01 | C*01:02 | C*12:03 |
| 17 | SCARs | DRESS | Cefazolin | Clinical | A*02:01 | A*33:03 | B*15:01 | B*44:03 | C*04:01 | C*07:06 |
| 18 | IR | Rash | Penicillin | SPT,IDT,DPT | A*24:07 | A*02:03 | B*58:01 | B*38:02 | C*07:02 | C*03:02 |
| 19 | IR | Angioedema | Augmentin | SPT,IDT | A*24:02 | A*33:03 | B*38:02 | B*58:01 | C*03:02 | C*07:02 |
| 20 | IR | Urticaria | Penicillin | SPT,IDT,DPT | A*24:03 | A*68:01 | B*15:01 | B*15:25 | C*04:03 | C*07:02 |
| 21 | IR | Angioedema | Augmentin | SPT,IDT,DPT | A*02:03 | A*02:07 | B*46:01 | B*46:01 | C*01:02 | C*01:02 |
| 22 | IR | Urticaria | Ceftriaxone | SPT | A*11:01 | A*24:07 | B*38:02 | B*46:01 | C*01:02 | C*08:01 |
| 23 | IR | Urticaria | Ceftriaxone | DPT | A*02:05 | A*11:01 | B*57:01 | B*40:06 | C*06:02 | C*15:02 |
| 24 | IR | Anaphylaxis | Tazocin | SPT | A*11:01 | A*11:01 | B*15:01 | B*27:04 | C*04:01 | C*12:02 |
| 25 | SCARs | AGEP | Ceftriaxone | Clinical | A*02:03 | A*02:03 | B*15:02 | B*38:02 | C*07:02 | C*08:01 |
| 26 | IR | Anaphylaxis | Augmentin | SPT,IDT,DPT | A*11:01 | A*33:03 | B*15:02 | B*58:01 | C*03:02 | C*08:01 |
| 27 | IR | Angioedema, urticaria | Augmentin | SPT,IDT | A*24:02 | A*26:01 | B*15:25 | B*52:01 | C*04:03 | C*07:02 |
| 28 | SCARs | SJS | Cephalexin | Clinical | A*11:01 | A*24:07 | B*15:02 | B*52:01 | C*08:01 | C*12:02 |
| 29 | SCARs | DRESS | Tazocin | Clinical | A*33:03 | A*11:01 | B*13:01 | B*58:01 | C*03:04 | C*03:02 |
| 30 | IR | Urticaria | Cephalosporin | SPT | A*33:03 | A*02:01 | B*40:01 | B*58:01 | C*07:02 | C*03:02 |
| 31 | IR | Anaphylaxis | Cefazolin | DPT | A*33:03 | A*33:03 | B*44:03 | B*58:01 | C*07:06 | C*03:02 |
| 32 | IR | Anaphylaxis | Ceftriaxone | DPT | A*24:02 | A*02:06 | B*40:01 | B*48:03 | C*08:01 | C*03:04 |
| 33 | SCARs | AGEP | Augmentin | Clinical, ELISPOT | A*01:01 | A*01:01 | B*57:01 | B*57:01 | C*06:02 | C*06:02 |
| 34 | IR | Anaphylaxis | Ceftriaxone | DPT | A*11:01 | A*02:07 | B*46:01 | B*15:02 | C*08:01 | C*01:02 |
| 35 | NIR-mild | Anaphylaxis | Ampicillin | DPT | A*24:07 | A*02:03 | B*39:09 | B*38:04 | C*07:02 | C*07:02 |
| 36 | IR | Anaphylaxis | Penicillin | SPT | A*11:01 | A*11:01 | B*40:01 | B*13:01 | C*07:02 | C*03:04 |
| 37 | IR | Anaphylaxis | Ceftriaxone | SPT,DPT | A*11:02 | A*33:03 | B*58:01 | B*46:01 | C*01:02 | C*03:02 |

**Abbreviations:** AGEP, acute generalized exanthematous pustulosis; DPT, drug provocation test; DRESS, drug reaction with eosinophilia and systemic symptoms; IDT, intradermal test; IR, immediate reaction; NIR-mild, non-immediate reaction with mild skin eruption; SCARs, severe cutaneous adverse reactions; SJS, Stevens–Johnson syndrome; SPT, skin prick test.

**Supplementary Table 2** HLA class II genotypes of 37 BL-HSR cases

| **ID** | **Reaction Type** | **Clinical** | **Drug** | **Confirm test** | **HLA- DRB1** | | **HLA-DQA1** | | **HLA-DQB1** | |
| --- | --- | --- | --- | --- | --- | --- | --- | --- | --- | --- |
| 1 | IR | Urticaria | Penicillin | SPT, DPT | DRB1*15:01 | DRB1*11:01 | DQA1*01:02 | DQA1*05:05 | DQB1*05:02 | DQB1*03:01 |
| 2 | IR | Angioedema | Amoxicillin | SPT, DPT | DRB1*15:02 | DRB1*09:01 | DQA1*01:01 | DQA1*03:02 | DQB1*03:03 | DQB1*05:01 |
| 3 | IR | Anaphylaxis | Penicillin | SPT | DRB1*15:02 | DRB1*12:02 | DQA1*01:01 | DQA1*06:01 | DQB1*03:01 | DQB1*05:01 |
| 4 | IR | Angioedema | Ampicillin,  Amoxicillin | SPT | DRB1*15:02 | DRB1*12:02 | DQA1*01:02 | DQA1*01:02 | DQB1*06:01 | DQB1*05:02 |
| 5 | NIR-mild | Fixed drug eruption | Penicillin | IDT | DRB1*09:01 | DRB1*08:03 | DQA1*01:03 | DQA1*03:02 | DQB1*06:01 | DQB1*03:03 |
| 6 | NIR-mild | MPE | Penicillin | SPT, DPT | DRB1*07:01 | DRB1*10:01 | DQA1*01:05 | DQA1*02:01 | DQB1*05:01 | DQB1*02:02 |
| 7 | NIR-mild | Angioedema, MPE | Amoxicillin,  Penicillin | SPT, DPT | DRB1*09:01 | DRB1*09:01 | DQA1*03:02 | DQA1*03:02 | DQB1*03:03 | DQB1*03:03 |
| 8 | NIR-mild | MPE | Ampicillin,  Penicillin | SPT,IDT | DRB1*15:02 | DRB1*09:01 | DQA1*01:01 | DQA1*03:02 | DQB1*03:03 | DQB1*05:01 |
| 9 | IR | Anaphylaxis | Amoxicillin | SPT,IDT,DPT | DRB1*15:02 | DRB1*11:06 | DQA1*01:01 | DQA1*05:05 | DQB1*03:01 | DQB1*05:01 |
| 10 | IR | Angioedema, urticaria | Penicillin | SPT,IDT | DRB1*09:01 | DRB1*08:03 | DQA1*01:03 | DQA1*03:02 | DQB1*06:01 | DQB1*03:03 |
| 11 | IR | Urticaria | Cloxacillin | SPT,IDT | DRB1*04:06 | DRB1*07:01 | DQA1*02:01 | DQA1*03:01 | DQB1*03:02 | DQB1*02:02 |
| 12 | NIR-mild | Angioedema | Augmentin | SPT,IDT,DPT | DRB1*15:02 | DRB1*09:01 | DQA1*01:01 | DQA1*03:02 | DQB1*03:03 | DQB1*05:01 |
| 13 | NIR-mild | Angioedema | Dicloxacillin | SPT,IDT,DPT | DRB1*08:03 | DRB1*12:02 | DQA1*01:03 | DQA1*06:01 | DQB1*06:01 | DQB1*03:01 |
| 14 | NIR-mild | MPE | Penicillin | SPT,IDT,DPT | DRB1*07:01 | DRB1*12:02 | DQA1*06:01 | DQA1*02:01 | DQB1*03:01 | DQB1*02:02 |
| 15 | IR | Urticaria | Cephalexin | SPT,IDT,DPT | DRB1*09:01 | DRB1*12:02 | DQA1*06:01 | DQA1*03:02 | DQB1*03:03 | DQB1*03:01 |
| 16 | SCARs | AGEP | Cefazolin | Clinical | DRB1*04:05 | DRB1*14:54 | DQA1*01:04 | DQA1*03:03 | DQB1*05:03 | DQB1*04:01 |
| 17 | SCARs | DRESS | Cefazolin | Clinical | DRB1*04:06 | DRB1*07:01 | DQA1*02:01 | DQA1*03:01 | DQB1*03:02 | DQB1*02:02 |
| 18 | IR | Rash | Penicillin | SPT,IDT,DPT | DRB1*04:05 | DRB1*10:01 | DQA1*01:05 | DQA1*03:03 | DQB1*05:01 | DQB1*04:01 |
| 19 | IR | Angioedema | Augmentin | SPT,IDT | DRB1*15:02 | DRB1*03:01 | DQA1*01:01 | DQA1*05:01 | DQB1*02:01 | DQB1*05:01 |
| 20 | IR | Urticaria | Penicillin | SPT,IDT,DPT | DRB1*15:02 | DRB1*15:02 | DQA1*01:01 | DQA1*01:01 | DQB1*05:01 | DQB1*05:01 |
| 21 | IR | Angioedema | Augmentin | SPT,IDT,DPT | DRB1*09:01 | DRB1*14:54 | DQA1*01:04 | DQA1*03:02 | DQB1*05:02 | DQB1*03:03 |
| 22 | IR | Urticaria | Ceftriaxone | SPT | DRB1*15:01 | DRB1*04:04 | DQA1*01:02 | DQA1*03:01 | DQB1*06:02 | DQB1*03:02 |
| 23 | IR | Urticaria | Ceftriaxone | DPT | DRB1*07:01 | DRB1*11:01 | DQA1*02:01 | DQA1*05:05 | DQB1*03:03 | DQB1*03:01 |
| 24 | IR | Anaphylaxis | Tazocin | SPT | DRB1*14:54 | DRB1*14:18 | DQA1*01:04 | DQA1*01:04 | DQB1*05:03 | DQB1*05:03 |
| 25 | SCARs | AGEP | Ceftriaxone | Clinical | DRB1*15:01 | DRB1*12:02 | DQA1*01:02 | DQA1*06:01 | DQB1*06:01 | DQB1*03:01 |
| 26 | IR | Anaphylaxis | Augmentin | SPT,IDT,DPT | DRB1*04:05 | DRB1*12:02 | DQA1*06:01 | DQA1*03:03 | DQB1*03:01 | DQB1*04:01 |
| 27 | IR | Angioedema, urticaria | Augmentin | SPT,IDT | DRB1*15:02 | DRB1*12:02 | DQA1*01:02 | DQA1*01:01 | DQB1*05:01 | DQB1*05:02 |
| 28 | SCARs | SJS | Cephalexin | Clinical | DRB1*04:03 | DRB1*15:02 | DQA1*01:01 | DQA1*03:01 | DQB1*03:02 | DQB1*05:01 |
| 29 | SCARs | DRESS | Tazocin | Clinical | DRB1*13:02 | DRB1*11:01 | DQA1*01:02 | DQA1*05:05 | DQB1*06:09 | DQB1*03:01 |
| 30 | IR | Urticaria | Cephalosporin | SPT | DRB1*04:05 | DRB1*03:01 | DQA1*05:01 | DQA1*03:03 | DQB1*03:02 | DQB1*02:01 |
| 31 | IR | Anaphylaxis | Cefazolin | DPT | DRB1*07:01 | DRB1*03:01 | DQA1*02:01 | DQA1*05:01 | DQB1*02:01 | DQB1*02:02 |
| 32 | IR | Anaphylaxis | Ceftriaxone | DPT | DRB1*11:01 | DRB1*11:01 | DQA1*05:05 | DQA1*05:05 | DQB1*03:01 | DQB1*03:01 |
| 33 | SCARs | AGEP | Augmentin | Clinical, ELISPOT | DRB1*07:01 | DRB1*07:01 | DQA1*02:01 | DQA1*02:01 | DQB1*03:03 | DQB1*03:03 |
| 34 | IR | Anaphylaxis | Ceftriaxone | DPT | DRB1*09:01 | DRB1*12:02 | DQA1*06:01 | DQA1*03:02 | DQB1*03:03 | DQB1*03:01 |
| 35 | NIR-mild | Anaphylaxis | Ampicillin | DPT | DRB1*08:02 | DRB1*12:02 | DQA1*01:02 | DQA1*04:01 | DQB1*05:02 | DQB1*04:02 |
| 36 | IR | Anaphylaxis | Penicillin | SPT | DRB1*04:06 | DRB1*09:01 | DQA1*03:01 | DQA1*03:02 | DQB1*03:03 | DQB1*03:02 |
| 37 | IR | Anaphylaxis | Ceftriaxone | SPT,DPT | DRB1*09:01 | DRB1*03:01 | DQA1*05:01 | DQA1*03:02 | DQB1*03:03 | DQB1*02:01 |

**Abbreviations:** AGEP, acute generalized exanthematous pustulosis; DPT, drug provocation test; DRESS, drug reaction with eosinophilia and systemic symptoms; IDT, intradermal test; IR, immediate reaction; NIR-mild, non-immediate reaction with mild skin eruption; SCARs, severe cutaneous adverse reactions; SJS, Stevens–Johnson syndrome; SPT, skin prick test.

**Supplementary Table 3** Association analysis of HLA-A alleles and BL-HSR

| **No.** | **HLA-A allele** | **Case exposed (2n)** | **Case non-exposed (2n)** | **Control exposed (2n)** | **Control non-exposed (2n)** | **Odds ratio** | **Lower bound 95% confidence interval** | **Upper bound 95% confidence interval** | **P-value** | **Adjusted p-value** |
| --- | --- | --- | --- | --- | --- | --- | --- | --- | --- | --- |
| 1 | A*01:01 | 2 | 72 | 49 | 2775 | 1.58 | 0.182 | 6.202 | 0.376 | 1 |
| 2 | A*02:01 | 4 | 70 | 88 | 2736 | 1.78 | 0.461 | 4.918 | 0.296 | 1 |
| 3 | A*02:03 | 7 | 67 | 291 | 2533 | 0.91 | 0.349 | 2.004 | 1 | 1 |
| 4 | A*02:05 | 1 | 73 | 3 | 2821 | 12.88 | 0.242 | 162.101 | 0.098 | 1 |
| 5 | A*02:06 | 1 | 73 | 42 | 2782 | 0.91 | 0.022 | 5.501 | 1 | 1 |
| 6 | A*02:07 | 7 | 67 | 304 | 2520 | 0.87 | 0.332 | 1.907 | 0.85 | 1 |
| 7 | A*11:01 | 16 | 58 | 496 | 2328 | 1.29 | 0.689 | 2.306 | 0.356 | 1 |
| 8 | A*11:02 | 1 | 73 | 49 | 2775 | 0.78 | 0.019 | 4.668 | 1 | 1 |
| 9 | A*24:02 | 8 | 66 | 201 | 2623 | 1.58 | 0.647 | 3.363 | 0.249 | 1 |
| 10 | A*24:03 | 1 | 73 | 13 | 2811 | 2.96 | 0.069 | 20.213 | 0.304 | 1 |
| 11 | A*24:07 | 4 | 70 | 88 | 2736 | 1.78 | 0.461 | 4.918 | 0.296 | 1 |
| 12 | A*24:17 | 1 | 73 | 2 | 2822 | 19.33 | 0.323 | 373.557 | 0.075 | 1 |
| 13 | A*26:01 | 2 | 72 | 28 | 2796 | 2.75 | 0.314 | 11.357 | 0.177 | 1 |
| 14 | A*30:01 | 1 | 73 | 30 | 2794 | 1.28 | 0.031 | 7.899 | 0.553 | 1 |
| 15 | A*31:01 | 2 | 72 | 35 | 2789 | 2.22 | 0.253 | 8.904 | 0.243 | 1 |
| 16 | A*33:03 | 13 | 61 | 193 | 2631 | 2.91 | 1.437 | 5.459 | **0.001** | **0.031** |
| 17 | A*34:01 | 1 | 73 | 18 | 2806 | 2.14 | 0.051 | 13.890 | 0.389 | 1 |
| 18 | A*68:01 | 2 | 72 | 19 | 2805 | 4.21 | 0.454 | 17.510 | 0.099 | 1 |

**Supplementary Table 4** Association analysis of HLA-B alleles and BL-HSR

| **No.** | **HLA-B allele** | **Case exposed (2n)** | **Case non-exposed (2n)** | **Control exposed (2n)** | **Control non-exposed (2n)** | **Odds ratio** | **Lower bound 95% confidence interval** | **Upper bound 95% confidence interval** | **P-value** | **Adjusted p-value** |
| --- | --- | --- | --- | --- | --- | --- | --- | --- | --- | --- |
| 1 | B*07:05 | 1 | 73 | 30 | 2892 | 1.32 | 0.032 | 8.175 | 0.541 | 1 |
| 2 | B*13:01 | 3 | 71 | 105 | 2817 | 1.13 | 0.225 | 3.543 | 0.749 | 1 |
| 3 | B*15:01 | 4 | 70 | 46 | 2876 | 3.57 | 0.908 | 10.185 | **0.034** | 0.968 |
| 4 | B*15:02 | 5 | 69 | 232 | 2690 | 0.84 | 0.262 | 2.086 | 1 | 1 |
| 5 | B*15:13 | 1 | 73 | 24 | 2898 | 1.65 | 0.040 | 10.431 | 0.466 | 1.000 |
| 6 | B*15:21 | 1 | 73 | 16 | 2906 | 2.49 | 0.059 | 16.436 | 0.347 | 1.000 |
| 7 | B*15:25 | 2 | 72 | 46 | 2876 | 1.74 | 0.200 | 6.869 | 0.333 | 1 |
| 8 | B*18:01 | 1 | 73 | 78 | 2844 | 0.50 | 0.012 | 2.956 | 0.722 | 1 |
| 9 | B*18:02 | 2 | 72 | 44 | 2878 | 1.82 | 0.209 | 7.204 | 0.315 | 1 |
| 10 | B*27:04 | 1 | 73 | 33 | 2889 | 1.20 | 0.029 | 7.375 | 0.575 | 1 |
| 11 | B*35:03 | 1 | 73 | 22 | 2900 | 1.81 | 0.043 | 11.483 | 0.439 | 1 |
| 12 | B*38:02 | 5 | 69 | 56 | 2866 | 3.71 | 1.124 | 9.591 | **0.016** | 0.464 |
| 13 | B*38:04 | 1 | 73 | 1 | 2921 | 40.01 | 0.503 | 3141.629 | **0.049** | 1.000 |
| 14 | B*39:01 | 1 | 73 | 21 | 2901 | 1.89 | 0.045 | 12.092 | 0.424 | 1.000 |
| 15 | B*39:09 | 1 | 73 | 29 | 2893 | 1.37 | 0.033 | 8.482 | 0.53 | 1.000 |
| 16 | B*40:01 | 7 | 67 | 187 | 2735 | 1.53 | 0.583 | 3.388 | 0.331 | 1 |
| 17 | B*40:06 | 3 | 71 | 36 | 2886 | 3.39 | 0.652 | 11.115 | 0.07 | 1 |
| 18 | B*44:03 | 3 | 71 | 108 | 2814 | 1.10 | 0.218 | 3.438 | 0.754 | 1 |
| 19 | B*46:01 | 9 | 65 | 372 | 2550 | 0.95 | 0.412 | 1.936 | 1 | 1 |
| 20 | B*48:03 | 1 | 73 | 10 | 2912 | 3.99 | 0.091 | 28.682 | 0.241 | 1.000 |
| 21 | B*51:01 | 2 | 72 | 64 | 2858 | 1.24 | 0.144 | 4.833 | 0.678 | 1 |
| 22 | B*51:02 | 1 | 73 | 27 | 2895 | 1.47 | 0.035 | 9.168 | 0.505 | 1.000 |
| 23 | B*52:01 | 2 | 72 | 63 | 2859 | 1.26 | 0.147 | 4.915 | 0.675 | 1 |
| 24 | B*54:01 | 3 | 71 | 29 | 2893 | 4.22 | 0.802 | 14.081 | **0.043** | 0.774 |
| 25 | B*55:01 | 1 | 73 | 4 | 2918 | 9.99 | 0.200 | 102.350 | 0.118 | 1.000 |
| 26 | B*56:01 | 1 | 73 | 19 | 2903 | 2.09 | 0.050 | 13.525 | 0.395 | 1.000 |
| 27 | B*56:04 | 1 | 73 | 17 | 2905 | 2.34 | 0.055 | 15.337 | 0.363 | 1.000 |
| 28 | B*57:01 | 3 | 71 | 28 | 2894 | 4.37 | 0.830 | 14.637 | **0.04** | 1.000 |
| 29 | B*58:01 | 7 | 67 | 204 | 2718 | 1.39 | 0.532 | 3.082 | 0.36 | 1 |

**Supplementary Table 5** Association analysis of HLA-C alleles and BL-HSR

| **No.** | **HLA-C allele** | **Case exposed (2n)** | **Case non-exposed (2n)** | **Control exposed (2n)** | **Control non-exposed (2n)** | **Odds ratio** | **Lower bound 95% confidence interval** | **Upper bound 95% confidence interval** | **P-value** | **Adjusted p-value** |
| --- | --- | --- | --- | --- | --- | --- | --- | --- | --- | --- |
| 1 | C*01:02 | 13 | 61 | 286 | 2538 | 1.89 | 0.941 | 3.532 | 0.050 | 0.75 |
| 2 | C*03:02 | 7 | 67 | 213 | 2611 | 1.28 | 0.490 | 2.833 | 0.503 | 1 |
| 3 | C*03:03 | 1 | 73 | 57 | 2767 | 0.66 | 0.016 | 3.977 | 1.000 | 1 |
| 4 | C*03:04 | 7 | 67 | 203 | 2621 | 1.35 | 0.516 | 2.987 | 0.491 | 1 |
| 5 | C*04:01 | 2 | 72 | 95 | 2729 | 0.80 | 0.093 | 3.071 | 1.000 | 1 |
| 6 | C*04:03 | 3 | 71 | 91 | 2733 | 1.27 | 0.251 | 3.982 | 0.733 | 1 |
| 7 | C*06:02 | 3 | 71 | 105 | 2719 | 1.09 | 0.217 | 3.420 | 0.755 | 1 |
| 8 | C*07:02 | 14 | 60 | 249 | 2575 | 2.41 | 1.227 | 4.445 | **0.006** | **0.097** |
| 9 | C*07:04 | 3 | 71 | 138 | 2686 | 0.82 | 0.164 | 2.555 | 1.000 | 1 |
| 10 | C*07:06 | 3 | 71 | 39 | 2785 | 3.02 | 0.583 | 9.845 | 0.090 | 1 |
| 11 | C*08:01 | 9 | 65 | 203 | 2621 | 1.79 | 0.771 | 3.674 | 0.111 | 1 |
| 12 | C*12:02 | 3 | 71 | 98 | 2726 | 1.18 | 0.233 | 3.680 | 0.743 | 1 |
| 13 | C*12:03 | 1 | 73 | 49 | 2775 | 0.78 | 0.019 | 4.668 | 1.000 | 1 |
| 14 | C*14:02 | 1 | 73 | 63 | 2761 | 0.60 | 0.015 | 3.577 | 1.000 | 1 |
| 15 | C*15:02 | 4 | 70 | 91 | 2733 | 1.72 | 0.445 | 4.746 | 0.305 | 1 |

**Supplementary Table 6** Association analysis of HLA-DRB1 alleles and BL-HSR

| **No.** | **HLA-DRB1 allele** | **Case exposed (2n)** | **Case non-exposed (2n)** | **Control exposed (2n)** | **Control non-exposed (2n)** | **Odds ratio** | **Lower bound 95% confidence interval** | **Upper bound 95% confidence interval** | **P-value** | **Adjusted p-value** |
| --- | --- | --- | --- | --- | --- | --- | --- | --- | --- | --- |
| 1 | DRB1*03:01 | 4 | 70 | 200 | 3484 | 1.00 | 0.261 | 2.703 | 1 | 1 |
| 2 | DRB1*04:03 | 1 | 73 | 70 | 3614 | 0.71 | 0.017 | 4.198 | 1 | 1 |
| 3 | DRB1*04:04 | 1 | 73 | 11 | 3673 | 4.57 | 0.105 | 32.209 | 0.213 |  |
| 4 | DRB1*04:05 | 4 | 70 | 183 | 3501 | 1.09 | 0.287 | 2.973 | 0.785 | 1 |
| 5 | DRB1*04:06 | 3 | 71 | 51 | 3633 | 3.01 | 0.587 | 9.661 | 0.089 | 1 |
| 6 | DRB1*07:01 | 8 | 66 | 237 | 3447 | 1.76 | 0.722 | 3.737 | 0.147 | 1 |
| 7 | DRB1*08:02 | 1 | 73 | 4 | 3680 | 12.60 | 0.252 | 129.060 | 0.095 | 1 |
| 8 | DRB1*08:03 | 3 | 71 | 57 | 3627 | 2.69 | 0.526 | 8.582 | 0.113 | 1 |
| 9 | DRB1*09:01 | 12 | 62 | 290 | 3394 | 2.27 | 1.098 | 4.304 | **0.016** | 0.272 |
| 10 | DRB1*10:01 | 2 | 72 | 75 | 3609 | 1.34 | 0.156 | 5.178 | 0.664 | 1 |
| 11 | DRB1*11:01 | 5 | 69 | 90 | 3594 | 2.89 | 0.889 | 7.328 | **0.038** | 0.646 |
| 12 | DRB1*11:06 | 1 | 73 | 63 | 3621 | 0.79 | 0.019 | 4.690 | 1 | 1 |
| 13 | DRB1*12:02 | 10 | 64 | 544 | 3140 | 0.90 | 0.410 | 1.782 | 0.869 | 1 |
| 14 | DRB1*13:02 | 1 | 73 | 41 | 3643 | 1.22 | 0.030 | 7.387 | 0.568 | 1 |
| 15 | DRB1*14:18 | 1 | 73 | 0 | 3684 | Exclude | NA | NA | NA | NA |
| 16 | DRB1*14:54 | 3 | 71 | 47 | 3637 | 3.27 | 0.636 | 10.541 | 0.074 | 1 |
| 17 | DRB1*15:01 | 3 | 71 | 282 | 3402 | 0.51 | 0.102 | 1.566 | 0.371 | 1 |
| 18 | DRB1*15:02 | 11 | 63 | 534 | 3150 | 1.03 | 0.486 | 1.987 | 0.868 | 1 |

**Abbreviations:** NA, not available.

**Supplementary Table 7** Association analysis of HLA-DQA1 alleles and BL hypersensitivity

| **No.** | **HLA-DQA1 allele** | **Case exposed (2n)** | **Case non-exposed (2n)** | **Control exposed (2n)** | **Control non-exposed (2n)** | **Odds ratio** | **Lower bound 95% confidence interval** | **Upper bound 95% confidence interval** | **P-value** | **Adjusted p-value** |
| --- | --- | --- | --- | --- | --- | --- | --- | --- | --- | --- |
| 1 | DQA1*01:01 | 10 | 64 | 425 | 1433 | 0.53 | 0.239 | 1.045 | 0.065 | 0.585 |
| 2 | DQA1*01:02 | 8 | 66 | 407 | 1451 | 0.43 | 0.178 | 0.912 | **0.021** | 0.189 |
| 3 | DQA1*01:03 | 3 | 71 | 69 | 1789 | 1.10 | 0.215 | 3.472 | 0.754 | 1 |
| 4 | DQA1*01:04 | 4 | 70 | 12 | 1846 | 8.79 | 2.010 | 29.866 | **0.003** | **0.029** |
| 5 | DQA1*01:05 | 2 | 72 | 0 | 1858 | Exclude | NA | NA | NA | NA |
| 6 | DQA1*02:01 | 8 | 66 | 142 | 1716 | 1.46 | 0.595 | 3.138 | 0.370 | 1 |
| 7 | DQA1*03:01 | 5 | 69 | 159 | 1699 | 0.77 | 0.240 | 1.935 | 0.831 | 1 |
| 8 | DQA1*03:02 | 12 | 62 | 160 | 1698 | 2.05 | 0.986 | 3.949 | **0.035** | 0.315 |
| 9 | DQA1*03:03 | 4 | 70 | 0 | 1858 | Exclude | NA | NA | NA | NA |
| 10 | DQA1*04:01 | 1 | 73 | 4 | 1854 | 6.35 | 0.127 | 65.055 | 0.178 | 1 |
| 11 | DQA1*05:01 | 4 | 70 | 168 | 1690 | 0.57 | 0.151 | 1.567 | 0.403 | 1 |
| 12 | DQA1*05:05 | 6 | 68 | 32 | 1826 | 5.03 | 1.662 | 12.740 | **0.003** | **0.029** |
| 13 | DQA1*06:01 | 7 | 67 | 203 | 1655 | 0.85 | 0.325 | 1.888 | 0.849 | 1 |

**Abbreviations:** NA, not available.

**Supplementary Table 8** Association analysis of HLA-DQB1 alleles and BL-HSR

| **No.** | **HLA-DQB1 allele** | **Case exposoed (2n)** | **Case non-exposed (2n)** | **Control exposed (2n)** | **Control non-exposed (2n)** | **Odds ratio** | **Lower bound 95% confidence interval** | **Upper bound 95% confidence interval** | **P-value** | **Adjusted p-value** |
| --- | --- | --- | --- | --- | --- | --- | --- | --- | --- | --- |
| 1 | DQB1*02:01 | 4 | 70 | 178 | 3280 | 1.05 | 0.276 | 2.865 | 0.791 | 1 |
| 2 | DQB1*02:02 | 5 | 69 | 140 | 3318 | 1.72 | 0.532 | 4.298 | 0.229 | 0.957 |
| 3 | DQB1*03:01 | 13 | 61 | 582 | 2876 | 1.05 | 0.527 | 1.953 | 0.875 | 1 |
| 4 | DQB1*03:02 | 6 | 68 | 159 | 3299 | 1.83 | 0.639 | 4.282 | 0.158 | 1 |
| 5 | DQB1*03:03 | 15 | 59 | 389 | 3069 | 2.01 | 1.046 | 3.622 | **0.025** | 0.253 |
| 6 | DQB1*04:01 | 3 | 71 | 131 | 3327 | 1.07 | 0.213 | 3.336 | 0.759 | 1 |
| 7 | DQB1*04:02 | 1 | 73 | 30 | 3428 | 1.57 | 0.038 | 9.689 | 0.483 |  |
| 8 | DQB1*05:01 | 12 | 62 | 443 | 3015 | 1.32 | 0.641 | 2.492 | 0.38 | 1 |
| 9 | DQB1*05:02 | 5 | 69 | 638 | 2820 | 0.32 | 0.100 | 0.789 | **0.009** | 0.099 |
| 10 | DQB1*05:03 | 3 | 71 | 183 | 3275 | 0.76 | 0.151 | 2.338 | 1 | 1 |
| 11 | DQB1*06:01 | 5 | 69 | 274 | 3184 | 0.84 | 0.263 | 2.086 | 1 | 1 |
| 12 | DQB1*06:02 | 1 | 73 | 38 | 3420 | 1.23 | 0.030 | 7.514 | 0.564 | 1 |
| 13 | DQB1*06:09 | 1 | 73 | 16 | 3442 | 2.95 | 0.069 | 19.464 | 0.303 | 1 |

**Supplementary Table 9** Association analysis of HLA-DPB1 alleles and BL-HSR

| **No.** | **HLA-DPB1 allele** | **Case exposed (2n)** | **Case non-exposed (2n)** | **Control exposed (2n)** | **Control non-exposed (2n)** | **Odds ratio** | **Lower bound 95% confidence interval** | **Upper bound 95% confidence interval** | **P-value** | **Adjusted p-value** |
| --- | --- | --- | --- | --- | --- | --- | --- | --- | --- | --- |
| 1 | DPB1*01:01 | 1 | 73 | 29 | 1531 | 0.72 | 0.017 | 4.493 | 1 | 1 |
| 2 | DPB1*02:01 | 9 | 65 | 125 | 1435 | 1.59 | 0.679 | 3.306 | 0.194 | 1 |
| 3 | DPB1*02:02 | 5 | 69 | 96 | 1464 | 1.11 | 0.340 | 2.797 | 0.803 | 1 |
| 4 | DPB1*03:01 | 10 | 64 | 118 | 1442 | 1.91 | 0.851 | 3.869 | 0.074 | 1 |
| 5 | DPB1*04:01 | 9 | 65 | 191 | 1369 | 0.99 | 0.427 | 2.045 | 1 | 1 |
| 6 | DPB1*04:02 | 1 | 73 | 51 | 1509 | 0.41 | 0.010 | 2.437 | 0.729 | 1 |
| 7 | DPB1*05:01 | 17 | 57 | 341 | 1219 | 1.07 | 0.574 | 1.888 | 0.775 | 1 |
| 8 | DPB1*09:01 | 1 | 73 | 10 | 1550 | 2.12 | 0.048 | 15.278 | 0.4 | 1 |
| 9 | DPB1*100:01 | 1 | 73 | 0 | 1560 | Exclude | NA | NA | NA | NA |
| 10 | DPB1*104:01 | 2 | 72 | 5 | 1555 | 8.64 | 0.807 | 53.682 | **0.037** | 0.592 |
| 11 | DPB1*107:01 | 1 | 73 | 12 | 1548 | 1.77 | 0.041 | 12.244 | 0.454 | 1 |
| 12 | DPB1*13:01 | 8 | 66 | 309 | 1251 | 0.49 | 0.201 | 1.039 | 0.069 | 0.385 |
| 13 | DPB1*135:01 | 1 | 73 | 8 | 1552 | 2.66 | 0.059 | 20.251 | 0.342 | 1 |
| 14 | DPB1*14:01 | 3 | 71 | 51 | 1509 | 1.25 | 0.244 | 4.018 | 0.733 | 1 |
| 15 | DPB1*14:02 | 1 | 73 | 0 | 1560 | Exclude | NA | NA | NA | NA |
| 16 | DPB1*17:01 | 1 | 73 | 24 | 1536 | 0.88 | 0.021 | 5.534 | 1 | 1 |
| 17 | DPB1*19:01 | 1 | 73 | 4 | 1556 | 5.33 | 0.107 | 54.609 | 0.207 | 1 |
| 18 | DPB1*21:01 | 2 | 72 | 48 | 1512 | 0.88 | 0.101 | 3.457 | 1 | 1 |

**Abbreviations:** NA, not available.

**Supplementary Table 10** Association of HLA haplotypes in patients with BL-HSR reactions and Thai population

| **No.** | **Haplotypes.** | **Case allele frequency (2n=74)** | **Control allele frequency**  **(2n=668)** | **Odds ratio** | **95%CI** | **Adjusted p-value*** |
| --- | --- | --- | --- | --- | --- | --- |
| **HLA-I 2 alleles (51 haplotypes)** | |  |  |  |  |  |
| 1 | B*46:01~C*01:02 | 0.1216 | 0.1110 | 1.20 | (0.357 - 3.201) | 1.000 |
| 2 | B*58:01~C*03:02 | 0.0946 | 0.0630 | 1.74 | (0.421 - 5.344) | 1.000 |
| 3 | A*02:07~B*46:01 | 0.0811 | 0.0650 | 1.24 | (0.232 - 4.284) | 1.000 |
| 4 | A*33:03~B*58:01 | 0.0811 | 0.0490 | 1.72 | (0.314 - 6.223) | 1.000 |
| 5 | A*33:03~C*03:02 | 0.0811 | 0.0500 | 1.62 | (0.296 - 5.791) | 1.000 |
| 6 | A*02:07~C*01:02 | 0.0755 | 0.0680 | 1.18 | (0.222 - 4.070) | 1.000 |
| 7 | B*15:02~C*08:01 | 0.0676 | 0.0760 | 1.09 | (0.205 - 3.699) | 1.000 |
| 8 | A*11:01~C*01:02 | 0.0596 | 0.0190 | 3.06 | (0.297 - 17.510) | 1.000 |
| 9 | B*38:02~C*07:02 | 0.0541 | 0.0210 | 2.62 | (0.261 - 14.105) | 1.000 |
| 10 | A*24:02~C*03:04 | 0.0541 | 0.0140 | 3.68 | (0.344 - 22.930) | 1.000 |
| 11 | A*11:01~B*13:01 | 0.0405 | 0.0200 | 2.59 | (0.257 - 13.907) | 1.000 |
| 12 | A*11:01~B*15:02 | 0.0405 | 0.0390 | 1.38 | (0.148 - 6.281) | 1.000 |
| 13 | A*33:03~B*44:03 | 0.0405 | 0.0540 | 0.99 | (0.109 - 4.268) | 1.000 |
| 14 | B*13:01~C*03:04 | 0.0405 | 0.0290 | 1.80 | (0.188 - 8.691) | 1.000 |
| 15 | B*40:01~C*03:04 | 0.0405 | 0.0230 | 2.26 | (0.229 - 11.606) | 1.000 |
| 16 | B*40:01~C*07:02 | 0.0405 | 0.0420 | 1.28 | (0.138 - 5.744) | 1.000 |
| 17 | B*44:03~C*07:06 | 0.0405 | 0.0580 | 0.94 | (0.104 - 4.008) | 1.000 |
| 18 | B*57:01~C*06:02 | 0.0405 | 0.0130 | 4.55 | (0.403 - 32.245) | 1.000 |
| 19 | A*02:03~C*07:02 | 0.0405 | 0.0290 | 1.80 | (0.188 - 8.691) | 1.000 |
| 20 | A*11:01~C*03:04 | 0.0405 | 0.0260 | 2.01 | (0.207 - 9.946) | 1.000 |
| 21 | A*33:03~C*07:06 | 0.0405 | 0.0530 | 0.99 | (0.109 - 4.268) | 1.000 |
| 22 | A*11:01~C*07:02 | 0.0270 | 0.0470 | 0.56 | (0.013 - 3.700) | 1.000 |
| 23 | A*01:01~B*57:01 | 0.0270 | 0.0010 | 9.14 | (0.115 - 718.204) | 1.000 |
| 24 | A*02:01~B*40:01 | 0.0270 | 0.0170 | 1.51 | (0.032 - 12.715) | 1.000 |
| 25 | A*02:03~B*38:02 | 0.0270 | 0.0100 | 3.04 | (0.057 - 38.287) | 1.000 |
| 26 | A*11:01~B*40:01 | 0.0270 | 0.0350 | 0.75 | (0.017 - 5.200) | 1.000 |
| 27 | B*15:25~C*04:03 | 0.0270 | 0.0240 | 1.13 | (0.025 - 8.630) | 1.000 |
| 28 | B*18:02~C*07:04 | 0.0270 | 0.0240 | 1.13 | (0.025 - 8.630) | 1.000 |
| 29 | A*01:01~C*06:02 | 0.0270 | 0.0150 | 1.82 | (0.038 - 16.535) | 1.000 |
| 30 | A*02:01~C*07:02 | 0.0270 | 0.0120 | 2.27 | (0.046 - 23.342) | 1.000 |
| 31 | A*02:03~C*01:02 | 0.0270 | 0.0160 | 1.82 | (0.038 - 16.535) | 1.000 |
| 32 | A*11:01~C*12:02 | 0.0270 | 0.0130 | 2.27 | (0.046 - 23.342) | 1.000 |
| 33 | A*24:02~C*07:02 | 0.0270 | 0.0230 | 1.13 | (0.025 - 8.630) | 1.000 |
| 34 | A*24:07~C*08:01 | 0.0270 | 0.0240 | 1.13 | (0.025 - 8.630) | 1.000 |
| 35 | A*11:01~C*08:01 | 0.0214 | 0.0400 | 0.69 | (0.016 - 4.724) | 1.000 |
| 36 | A*02:03~B*46:01 | 0.0135 | 0.0120 | 2.24 | (0.045 - 23.023) | 1.000 |
| 37 | A*11:01~B*27:04 | 0.0135 | 0.0120 | 2.24 | (0.045 - 23.023) | 1.000 |
| 38 | A*11:01~B*51:01 | 0.0135 | 0.0120 | 2.24 | (0.045 - 23.023) | 1.000 |
| 39 | A*11:02~B*46:01 | 0.0135 | 0.0140 | 1.79 | (0.037 - 16.308) | 1.000 |
| 40 | B*15:13~C*08:01 | 0.0135 | 0.0130 | 2.24 | (0.045 - 23.023) | 1.000 |
| 41 | B*18:01~C*07:04 | 0.0135 | 0.0150 | 1.79 | (0.037 - 16.308) | 1.000 |
| 42 | B*27:04~C*12:02 | 0.0135 | 0.0120 | 2.24 | (0.045 - 23.023) | 1.000 |
| 43 | B*39:01~C*07:02 | 0.0135 | 0.0120 | 2.24 | (0.045 - 23.023) | 1.000 |
| 44 | B*39:09~C*07:02 | 0.0135 | 0.0160 | 1.79 | (0.037 - 16.308) | 1.000 |
| 45 | B*51:01~C*14:02 | 0.0135 | 0.0100 | 3.00 | (0.056 - 37.765) | 1.000 |
| 46 | B*51:02~C*15:02 | 0.0135 | 0.0150 | 1.79 | (0.037 - 16.308) | 1.000 |
| 47 | B*52:01~C*07:02 | 0.0135 | 0.0130 | 2.24 | (0.045 - 23.023) | 1.000 |
| 48 | B*52:01~C*12:02 | 0.0135 | 0.0100 | 3.00 | (0.056 - 37.765) | 1.000 |
| 49 | A*11:01~C*15:02 | 0.0135 | 0.0130 | 2.24 | (0.045 - 23.023) | 1.000 |
| 50 | A*11:01~C*14:02 | 0.0135 | 0.0160 | 1.82 | (0.038 - 16.535) | 1.000 |
| **HLA-II 2 alleles (41 haplotypes)** | |  |  |  |  |  |
| 51 | DRB1*09:01~DQB1*03:03 | 0.1622 | 0.1060 | 1.60 | (0.529 - 4.019) | 1.000 |
| 52 | DRB1*15:02~DQB1*05:01 | 0.1351 | 0.0780 | 1.79 | (0.519 - 4.941) | 1.000 |
| 53 | DRB1*09:01~DPB1*05:01 | 0.1081 | 0.0570 | 1.95 | (0.469 - 6.098) | 1.000 |
| 54 | DRB1*12:02~DQB1*03:01 | 0.0946 | 0.1180 | 0.92 | (0.232 - 2.668) | 1.000 |
| 55 | DQB1*03:03~DPB1*05:01 | 0.0831 | 0.0620 | 1.30 | (0.243 - 4.521) | 1.000 |
| 56 | DQB1*05:01~DPB1*13:01 | 0.0811 | 0.0580 | 1.44 | (0.267 - 5.080) | 1.000 |
| 57 | DRB1*15:02~DPB1*13:01 | 0.0811 | 0.0620 | 1.30 | (0.243 - 4.521) | 1.000 |
| 58 | DRB1*07:01~DQB1*02:02 | 0.0676 | 0.0790 | 1.04 | (0.197 - 3.536) | 1.000 |
| 59 | DQB1*03:01~DPB1*05:01 | 0.0623 | 0.0330 | 1.66 | (0.175 - 7.820) | 1.000 |
| 60 | DRB1*12:02~DPB1*05:01 | 0.0541 | 0.0140 | 3.68 | (0.344 - 22.930) | 1.000 |
| 61 | DQB1*02:01~DPB1*04:01 | 0.0541 | 0.0340 | 1.66 | (0.175 - 7.820) | 1.000 |
| 62 | DRB1*03:01~DPB1*04:01 | 0.0541 | 0.0340 | 1.66 | (0.175 - 7.820) | 1.000 |
| 63 | DRB1*03:01~DQB1*02:01 | 0.0541 | 0.0400 | 1.40 | (0.150 - 6.370) | 1.000 |
| 64 | DQB1*03:01~DPB1*03:01 | 0.0458 | 0.0260 | 2.03 | (0.210 - 10.088) | 1.000 |
| 65 | DQB1*05:01~DPB1*04:01 | 0.0405 | 0.0100 | 6.07 | (0.497 - 53.670) | 1.000 |
| 66 | DRB1*04:05~DQB1*04:01 | 0.0405 | 0.0310 | 1.80 | (0.188 - 8.691) | 1.000 |
| 67 | DRB1*04:06~DQB1*03:02 | 0.0405 | 0.0190 | 3.02 | (0.293 - 17.266) | 1.000 |
| 68 | DRB1*07:01~DQB1*03:03 | 0.0405 | 0.0130 | 4.55 | (0.403 - 32.245) | 1.000 |
| 69 | DRB1*08:03~DQB1*06:01 | 0.0405 | 0.0210 | 2.59 | (0.257 - 13.907) | 1.000 |
| 70 | DRB1*12:02~DQB1*05:02 | 0.0405 | 0.0290 | 1.80 | (0.188 - 8.691) | 1.000 |
| 71 | DQB1*03:01~DPB1*02:01 | 0.0405 | 0.0160 | 1.82 | (0.038 - 16.535) | 1.000 |
| 72 | DRB1*12:02~DPB1*02:01 | 0.0405 | 0.0210 | 1.29 | (0.028 - 10.294) | 1.000 |
| 73 | DQB1*03:03~DPB1*02:02 | 0.0385 | 0.0120 | 2.27 | (0.046 - 23.342) | 1.000 |
| 74 | DQB1*03:03~DPB1*13:01 | 0.0270 | 0.0160 | 1.82 | (0.038 - 16.535) | 1.000 |
| 75 | DRB1*07:01~DPB1*03:01 | 0.0270 | 0.0150 | 1.82 | (0.038 - 16.535) | 1.000 |
| 76 | DRB1*09:01~DPB1*02:02 | 0.0270 | 0.0100 | 3.04 | (0.057 - 38.287) | 1.000 |
| 77 | DRB1*10:01~DQB1*05:01 | 0.0270 | 0.0210 | 1.29 | (0.028 - 10.294) | 1.000 |
| 78 | DRB1*14:54~DQB1*05:03 | 0.0270 | 0.0100 | 3.04 | (0.057 - 38.287) | 1.000 |
| 79 | DQB1*02:02~DPB1*03:01 | 0.0218 | 0.0120 | 2.27 | (0.046 - 23.342) | 1.000 |
| 80 | DRB1*15:01~DPB1*02:01 | 0.0135 | 0.0130 | 2.27 | (0.046 - 23.342) | 1.000 |
| 81 | DQB1*03:01~DPB1*21:01 | 0.0135 | 0.0130 | 2.24 | (0.045 - 23.023) | 1.000 |
| 82 | DQB1*05:02~DPB1*04:01 | 0.0135 | 0.0110 | 2.24 | (0.045 - 23.023) | 1.000 |
| 83 | DQB1*05:02~DPB1*05:01 | 0.0135 | 0.0560 | 0.462 | (0.011 - 2.992) | 1.000 |
| 84 | DRB1*04:05~DPB1*03:01 | 0.0135 | 0.0130 | 2.24 | (0.045 - 23.023) | 1.000 |
| 85 | DRB1*07:01~DPB1*13:01 | 0.0135 | 0.0120 | 2.24 | (0.045 - 23.023) | 1.000 |
| 86 | DRB1*04:03~DQB1*03:02 | 0.0135 | 0.0190 | 1.49 | (0.032 - 12.541) | 1.000 |
| 87 | DRB1*04:05~DQB1*03:02 | 0.0135 | 0.0120 | 2.24 | (0.045 - 23.023) | 1.000 |
| 88 | DRB1*13:02~DQB1*06:09 | 0.0135 | 0.0130 | 2.24 | (0.045 - 23.023) | 1.000 |
| 89 | DRB1*14:54~DQB1*05:02 | 0.0135 | 0.0580 | 0.46 | (0.011 - 2.992) | 1.000 |
| 90 | DRB1*15:01~DQB1*05:02 | 0.0135 | 0.0250 | 1.11 | (0.025 - 8.511) | 1.000 |
| 91 | DRB1*15:01~DQB1*06:01 | 0.0135 | 0.0510 | 0.52 | (0.012 - 3.400) | 1.000 |
| **HLA-I 3 alleles (9 haplotypes)** | |  |  |  |  |  |
| 92 | A*02:07~B*46:01~C*01:02 | 0.0811 | **0.0650** | 1.24 | (0.232 - 4.284) | 1.000 |
| 93 | A*33:03~B*58:01~C*03:02 | 0.0811 | **0.0490** | 1.72 | (0.314 - 6.223) | 1.000 |
| 94 | A*11:01~B*13:01~C*03:04 | 0.0405 | **0.0150** | 3.68 | (0.344 - 22.930) | 1.000 |
| 95 | A*11:01~B*15:02~C*08:01 | 0.0405 | **0.0360** | 1.52 | (0.162 - 7.023) | 1.000 |
| 96 | A*01:01~B*57:01~C*06:02 | 0.0270 | **0.0100** | 3.04 | (0.057 - 38.287) | 1.000 |
| 97 | A*02:03~B*38:02~C*07:02 | 0.0270 | **0.0100** | 3.04 | (0.057 - 38.287) | 1.000 |
| 98 | A*11:01~B*40:01~C*07:02 | 0.0270 | **0.0210** | 1.29 | (0.028 - 10.294) | 1.000 |
| 99 | A*02:03~B*46:01~C*01:02 | 0.0135 | **0.0130** | 2.27 | (0.046 - 23.342) | 1.000 |
| 100 | A*11:01~B*51:01~C*14:02 | 0.0135 | **0.0150** | 1.82 | (0.038 - 16.535) | 1.000 |
| **HLA-II 3 alleles (7 haplotypes)** | |  |  |  |  |  |
| 101 | DRB1*09:01~DQB1*03:03~DPB1*05:01 | 0.1081 | 0.0590 | 1.85 | (0.447 - 5.741) | 1.000 |
| 102 | DRB1*15:02~DQB1*05:01~DPB1*13:01 | 0.0811 | 0.0590 | 1.37 | (0.254 - 4.785) | 1.000 |
| 103 | DRB1*03:01~DQB1*02:01~DPB1*04:01 | 0.0541 | 0.0340 | 1.66 | (0.175 - 7.820) | 1.000 |
| 104 | DRB1*12:02~DQB1*03:01~DPB1*05:01 | 0.0405 | 0.0170 | 3.06 | (0.297 - 17.510) | 1.000 |
| 105 | DRB1*07:01~DQB1*02:02~DPB1*03:01 | 0.0270 | 0.0130 | 2.27 | (0.046 - 23.342) | 1.000 |
| 106 | DRB1*12:02~DQB1*03:01~DPB1*02:01 | 0.0270 | 0.0170 | 1.51 | (0.032 - 12.715) | 1.000 |
| 107 | DRB1*12:02~DQB1*03:01~DPB1*13:01 | 0.0135 | 0.0110 | 2.27 | (0.046 - 23.342) | 1.000 |
| **HLA 4 alleles (4 haplotypes)** | |  |  |  |  |  |
| 108 | A*02:07~B*46:01~C*01:02~DRB1*09:01 | 0.0676 | 0.0320 | 2.52 | (0.441 - 9.843) | 0.623 |
| 109 | A*33:03~B*58:01~C*03:02~DRB1*03:01 | 0.0541 | 0.0240 | 2.29 | (0.232 - 11.771) | 1.000 |
| 110 | A*33:03~B*44:03~C*07:06~DRB1*07:01 | 0.0405 | 0.0420 | 1.30 | (0.140 - 5.826) | 1.000 |
| 111 | A*02:07~B*46:01~C*01:02~DRB1*12:02 | 0.0135 | 0.0100 | 3.04 | (0.057 - 38.287) | 1.000 |
| **HLA 5 alleles (2 haplotypes)** | |  |  |  |  |  |
| 112 | A*02:07~B*46:01~C*01:02~DRB1*09:01~DQB1*03:03 | 0.0676 | 0.0370 | 2.31 | (0.408 - 8.828) | 0.363 |
| 113 | A*33:03~B*58:01~C*03:02~DRB1*03:01~DQB1*02:01 | 0.0541 | 0.0210 | 2.62 | (0.261 - 14.105) | 0.448 |
| **HLA 6 alleles (2 haplotypes)** | |  |  |  |  |  |
| 114 | A*33:03~B*58:01~C*03:02~DRB1*03:01~DQB1*02:01~DPB1*04:01 | 0.0541 | 0.0190 | 3.06 | (0.297 - 17.510) | 0.371 |
| 115 | A*02:07~B*46:01~C*01:02~DRB1*09:01~DQB1*03:03~DPB1*05:01 | 0.0405 | 0.0190 | 3.06 | (0.297 - 17.510) | 0.371 |

* p-value was adjusted using Bonferroni correction for each haplotype number groups
